# Integrative Genomics in Combination with RNA Interference Identifies Prognostic and Functionally Relevant Gene Targets for Oral Squamous Cell Carcinoma

**DOI:** 10.1371/journal.pgen.1003169

**Published:** 2013-01-17

**Authors:** Chang Xu, Pei Wang, Yan Liu, Yuzheng Zhang, Wenhong Fan, Melissa P. Upton, Pawadee Lohavanichbutr, John R. Houck, David R. Doody, Neal D. Futran, Lue Ping Zhao, Stephen M. Schwartz, Chu Chen, Eduardo Méndez

**Affiliations:** 1 Department of Otolaryngology–Head and Neck Surgery, University of Washington, Seattle, Washington, United States of America; 2 Clinical Research Division, Fred Hutchinson Cancer Research Center, Seattle, Washington, United States of America; 3 Public Health Sciences Division, Fred Hutchinson Cancer Research Center, Seattle, Washington, United States of America; 4 Department of Biostatistics, University of Washington, Seattle, Washington, United States of America; 5 Department of Pathology, University of Washington, Seattle, Washington, United States of America; 6 Department of Epidemiology, University of Washington, Seattle, Washington, United States of America; 7 Surgery and Perioperative Care Service, VA Puget Sound Health Care System, Seattle, Washington, United States of America; Stanford University School of Medicine, United States of America

## Abstract

In oral squamous cell carcinoma (OSCC), metastasis to lymph nodes is associated with a 50% reduction in 5-year survival. To identify a metastatic gene set based on DNA copy number abnormalities (CNAs) of differentially expressed genes, we compared DNA and RNA of OSCC cells laser-microdissected from non-metastatic primary tumors (n = 17) with those from lymph node metastases (n = 20), using Affymetrix 250K Nsp single-nucleotide polymorphism (SNP) arrays and U133 Plus 2.0 arrays, respectively. With a false discovery rate (FDR)<5%, 1988 transcripts were found to be differentially expressed between primary and metastatic OSCC. Of these, 114 were found to have a significant correlation between DNA copy number and gene expression (FDR<0.01). Among these 114 correlated transcripts, the corresponding genomic regions of each of 95 transcripts had CNAs differences between primary and metastatic OSCC (FDR<0.01). Using an independent dataset of 133 patients, multivariable analysis showed that the OSCC–specific and overall mortality hazards ratio (HR) for patients carrying the 95-transcript signature were 4.75 (95% CI: 2.03–11.11) and 3.45 (95% CI: 1.84–6.50), respectively. To determine the degree by which these genes impact cell survival, we compared the growth of five OSCC cell lines before and after knockdown of over-amplified transcripts via a high-throughput siRNA–mediated screen. The expression-knockdown of 18 of the 26 genes tested showed a growth suppression ≥30% in at least one cell line (P<0.01). In particular, cell lines derived from late-stage OSCC were more sensitive to the knockdown of G3BP1 than cell lines derived from early-stage OSCC, and the growth suppression was likely caused by increase in apoptosis. Further investigation is warranted to examine the biological role of these genes in OSCC progression and their therapeutic potentials.

## Author Summary

Neck lymph node metastasis is the most important prognostic factor in oral squamous cell carcinoma (OSCC). To identify genes associated with this critical step of OSCC progression, we compared DNA copy number aberrations and gene expression differences between tumor cells found in metastatic lymph nodes versus those in non-metastatic primary tumors. We identified 95 transcripts (87 genes) with metastasis-specific genome abnormalities and gene expression. Tested in an independent cohort of 133 OSCC patients, the 95 gene signature was an independent risk factor of disease-specific and overall death, suggesting a disease progression phenotype. We knocked down the expression of over-amplified genes in five OSCC cell lines. Knockdown of 18 of the 26 tested genes suppressed the cell growth in at least one cell line. Interestingly, cell lines derived from late-stage OSCC were more sensitive to the knockdown of G3BP1 than cell lines derived from early-stage OSCC. The knockdown of G3BP1 increased programmed cell death in the p53-mutant but not wild-type OSCC cell lines. Taken together, we demonstrate that CNA–associated transcripts differentially expressed in carcinoma cells with an aggressive phenotype (i.e., metastatic to lymph nodes) can be biomarkers with both prognostic information and functional relevance. Moreover, results suggest that G3BP1 is a potential therapeutic target against late-stage p53-negative OSCC.

## Introduction

Metastatic spread to the cervical lymph nodes is a major feature associated with tumor aggressiveness in oral squamous cell carcinoma (OSCC), reducing 5-year survival by about 50% [1]–[3]. For these patients, treatment intensification with radiation and chemotherapy results in improved survival outcomes Cooper [4]. However, toxicities from such treatments can be severe, often resulting in life-long swallowing and speech impairments [5], and those for whom treatment fails invariably die of their disease. Unfortunately, despite recent advances in less toxic, targeted therapies, the treatment choices for advanced stage OSCC remain broad-based and do not target specific tumor biology. There is an urgent need to better understand the mechanism underlying the lymphotropism of OSCC tumor cells and to develop specific, less toxic therapies to target this event.

An important step in the development of targeted therapies is to identify the genes that are responsible for the initiation and progression of the disease. DNA microarray-based transcriptome profiling has been proven an effective tool for the identification of candidate biomarkers and therapeutic targets in solid tumors including OSCC. However, based on the transcriptome profiles alone it is challenging to determine whether expression changes for a given gene are causal or merely a consequence of a disease process. On the other hand, most human cancers display genome instability. Genome regions with recurrent aberrations are believed to harbor oncogenes or tumor suppressor genes that are essential for cancer initiation and progression. Previous studies have shown that combining DNA copy number aberrations (CNAs) and differential gene expression (i.e., “integrative genomics”) has helped oncogene discovery [6]–[8] and creation of better prognostic models [9]–[12].

In this study, we focused on metastasis by comparing the genomic and transcriptomic profiles of tumor cells laser-microdissected from metastatic lymph nodes to those from non-metastatic primary carcinomas. We report our efforts to identify the genes with CNAs that are both associated with differential gene expression and also unique to metastatic tumor cells. Furthermore, reasoning that some CNAs-associated transcripts differentially expressed in OSCC metastatic tumor cells could be driver oncogenes that confer to cells a disease progression phenotype, we performed a large-scale siRNA interference screen to identify those genes with high impact on cell growth and survival.

## Results

### Study population

Selected characteristics of the study participants are shown in Table S1. Among the 20 OSCC patients with lymph node metastasis, eight had cancers arising in the oropharynx (including 5 that were HPV 16+), while the remaining carcinomas arose from the oral cavity. The majority of patients had ≥N2 nodal staging (i.e. multiple metastatic nodes detected). The age range of the patients with metastases was 23–84 (mean 56.8) years. Of the 17 non-metastatic primary OSCC patients, three had primary tumors that arose in the oropharynx (including one that was HPV 16+), and the remaining tumors were from the oral cavity. Eight of these patients with non-metastatic primary OSCC had AJCC stage I tumors, and nine had AJCC stage II tumors. The age range of patients without metastases was 47–79 (mean 59.2) years. The mean follow-up interval for the patients with non-metastatic primaries was 2.7 years (range: 0.5–5.4 years), and none had nodal disease diagnosed during this period.

### Determining CNA–associated transcripts differentially expressed in tumor cells from metastatic lymph nodes

CNAs were detected in all 37 OSCC and on all chromosomal arms that were covered with SNP probes (short chromosomal arms 13p, 14p, 15p, 21p and 22p are not covered by the 250k Nsp SNP array). For each probe, the percentage of samples showing CNA in the 17 non-metastatic primary OSCC and the 20 nodal metastases is 54.4±16.8% (mean ± s.d.) and 39.79±15.51%, respectively (Figures S1 and S2).

**Figure 1:**
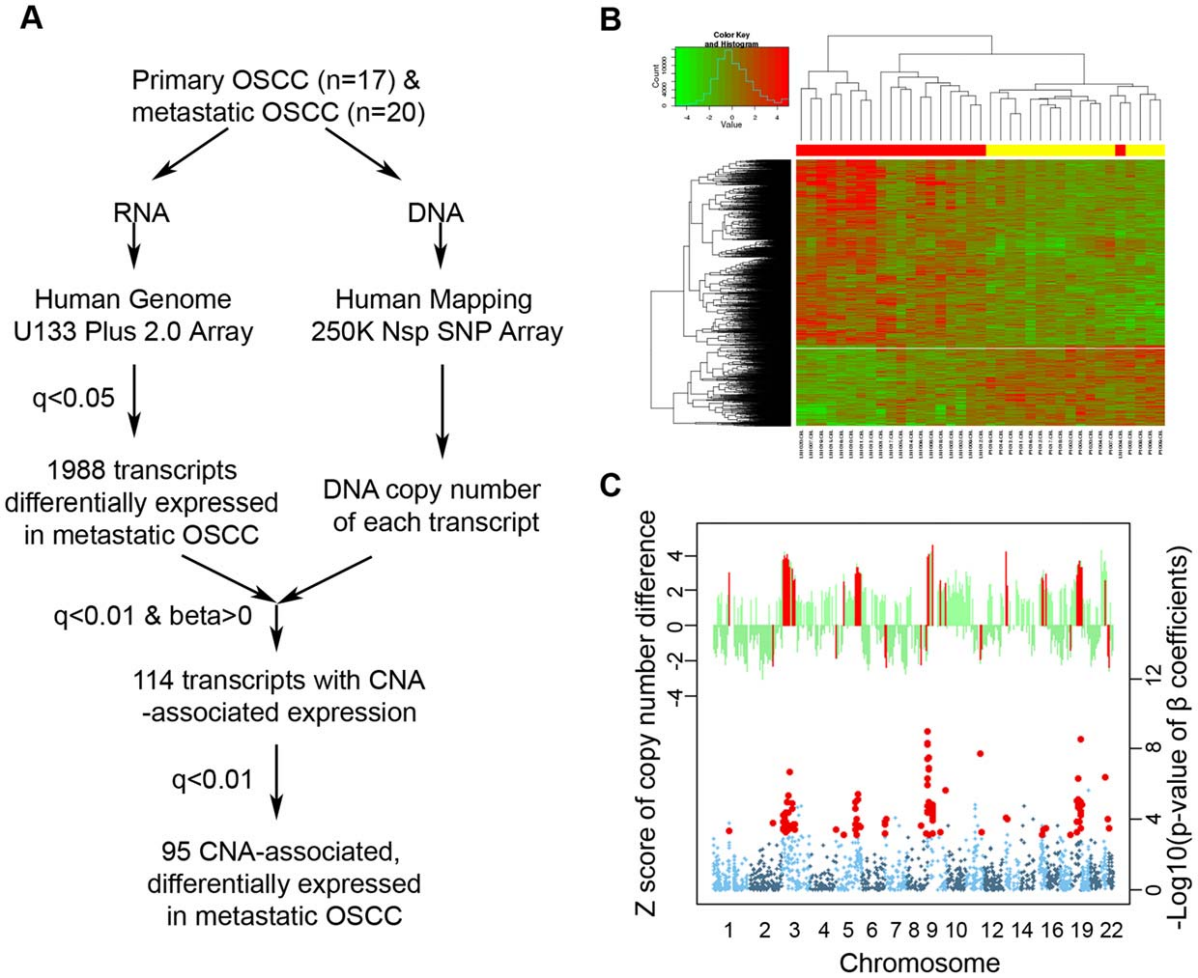
Identification of DNA copy number–associated differentially expressed genes in metastatic OSCC. A) Integrative genomic analysis workflow; B) Heat-map of the 1,988 transcripts with differential expression between non-metastatic primary and nodal metastatic OSCC. Specimen code: Red = metastatic OSCC; Yellow: primary OSCC; C) Top panel: the z-score of the DNA copy number difference between primary and metastatic OSCC. Each bar represents one of the differentially expressed genes. Genes with significant Z-score differences are highlighted in red. Bottom panel: Manhattan plot showing the -Log10 transformed p-value of the correlation coefficients of CNA and gene expression. Each dot represents one of the differentially expressed genes. Genes with different copy number in primary and metastatic OSCC are highlighted in red. The 4 regions with cluster of CNA-associated differentially expressed genes are indicated.

A flowchart of our integrative genomic analysis strategy is shown in Figure 1A. With a false positive rate <5%, 1,988 transcripts (representing 1,422 known genes and 306 unique unknown transcripts) were found to be differentially expressed between tumor cells from lymph node metastases and those from node-negative primary carcinomas (Figure 1B). We established the genome DNA copy number of 1,985 of the 1,988 transcripts (three transcripts had no SNP within their 250 kb neighboring region). Among these, 114 were found to have a significant correlation between DNA copy number and gene expression (FDR<0.01, Figure 1C, bottom panel), and of these 114 correlated transcripts, the corresponding genomic regions of each of 95 transcripts (representing 73 known genes and 14 unique unknown transcripts) had CNAs differences between metastatic and non-metastatic tumor cells (FDR<0.01, Figure 1C, top panel). Fifty-nine of these 95 transcripts, Table 1) were located in the following four chromosomal regions: 3p25.3-21.1, 5q31-35.3, 9p24.3- 22.3, and 18q21-23. IPA analysis categorized the 73 known genes with the following molecular and cellular functions: “Cell Death” (21 genes), “Gene Expression” (16 genes), and “Cell Cycle” (11 genes) (Table S2).

**Table 1:** DNA copy number–associated and differentially expressed transcripts in metastatic OSCC.

Gene	Probe Set	Gene Title	Cytoband
**Genes with higher DNA copy number and expression in metastatic OSCC**			
TRIM33	212436_at	tripartite motif-containing 33	1p13.1
RAD18	238670_at 223417_at	RAD18 homolog (S. cerevisiae)	3p25-p24
—	1557238_s_at	—	3p25.3
RAF1	1557675_at	V-raf-1 murine leukemia viral oncogene homolog 1	3p25
—	214116_at	—	3p25.1
OXNAD1	227686_at	oxidoreductase NAD-binding domain containing 1	3p25-p24
NGLY1	220742_s_at	N-glycanase 1	3p24.2
EOMES	231776_at	eomesodermin homolog (Xenopus laevis)	3p21.3-p21.2
AZI2	222498_at	5-azacytidine induced 2	3p24.1
CRTAP	201380_at	cartilage associated protein	3p22.3
—	236375_at	—	3p22.2
ZNF621	1558620_at	zinc finger protein 621	3p22.1
CTNNB1	223679_at	catenin (cadherin-associated protein), beta 1, 88 kDa	3p21
SNRK	207474_at	SNF related kinase	3p22.1
—	244145_at	—	3p21.31
USP4	202682_s_at	ubiquitin specific peptidase 4 (proto-oncogene)	3p21.3
ACTR8	218658_s_at	ARP8 actin-related protein 8 homolog (yeast)	3p21.1
FOXP1	235444_at	forkhead box P1	3p14.1
—	244845_at	—	3p13
FLJ10213	219906_at	endogenous Borna-like N element-1	3p13
—	1558714_at	—	3p12.3
CGGBP1	224599_at	CGG triplet repeat binding protein 1	3p12-p11.1
—	1556743_at	—	3p11.1
PPAP2A	209147_s_at	phosphatidic acid phosphatase type 2A	5q11
UBE2D2	201344_at	ubiquitin-conjugating enzyme E2D 2 (UBC4/5 homolog, yeast)	5q31.2
ANKHD1	229457_at	ankyrin repeat and KH domain containing 1	5q31.3
TMCO6	213550_s_at	transmembrane and coiled-coil domains 6	5q31.3
NDUFA2	209224_s_at	NADH dehydrogenase (ubiquinone) 1 alpha subcomplex, 2, 8 kDa	5q31
KIAA0141	227056_at	KIAA0141	5q31.3
NDFIP1	222422_s_at	Nedd4 family interacting protein 1	5q31.3
FBXO38	221257_x_at	F-box protein 38	5q32
PCYOX1L	218953_s_at	prenylcysteine oxidase 1 like	5q32
G3BP1	1557350_at	GTPase activating protein (SH3 domain) binding protein 1	5q33.1
MRPL22	218339_at	mitochondrial ribosomal protein L22	5q33.1-q33.3
CCNG1	208796_s_at	cyclin G1	5q32-q34
DOK3	223553_s_at	docking protein 3	5q35.3
—	241756_at	—	9p24.3
—	217671_at	—	9p24.2
C9orf46	218992_at	chromosome 9 open reading frame 46	9p24.1
CD274	227458_at	CD274 molecule	9p24
KIAA1432	226221_at 226222_at	KIAA1432	9p24.1
KIAA2026	228446_at	KIAA2026	9p24.1
UHRF2	225610_at	ubiquitin-like with PHD and ring finger domains 2	9p24.1
KDM4C	214861_at	lysine (K)-specific demethylase 4C	9p24.1
SNAPC3	204001_at 222286_at	small nuclear RNA activating complex, polypeptide 3, 50 kDa	9p22.3
PSIP1	209337_at	PC4 and SFRS1 interacting protein 1	9p22.3
—	232363_at	—	9p22.3
—	232681_at	—	9p22.3
LOC401504	226635_at	Hypothetical gene supported by AK091718	9p13.2
—	234032_at	—	9p13.2
ZCCHC7	230332_at 226496_at	Zinc finger, CCHC domain containing 7	9p13.2
—	1556543_at	—	9p13.2
EXOSC3	233495_at	exosome component 3	9p11
RG9MTD3	240166_x_at	RNA (guanine-9-) methyltransferase domain containing 3	9p13.2
DCAF10	230679_at	DDB1 and CUL4 associated factor 10	9p13.2
PTPDC1	229517_at	protein tyrosine phosphatase domain containing 1	9q22.32
PPP6C	225429_at	protein phosphatase 6, catalytic subunit	9q33.3
SAP18	208740_at	Sin3A-associated protein, 18 kDa	13q12.11
PAN3	225563_at	PAN3 poly(A) specific ribonuclease subunit homolog (S. cerevisiae)	13q12.2
FAM96A	224779_s_at	family with sequence similarity 96, member A	15q22.31
PIAS1	217863_at	protein inhibitor of activated STAT, 1	15q
CRTC3	218648_at	CREB regulated transcription coactivator 3	15q26.1
KIAA1632	232030_at	KIAA1632	18q12.3-q21.1
HAUS1	225297_at	HAUS augmin-like complex, subunit 1	18q21.1
SMAD2	226563_at	SMAD family member 2	18q21.1
SMAD4	202526_at 235725_at	SMAD family member 4	18q21.1
TXNL1	235561_at	thioredoxin-like 1	18q21.31
WDR7	212880_at	WD repeat domain 7	18q21.1-q22
MALT1	210017_at	mucosa associated lymphoid tissue lymphoma translocation gene 1	18q21
—	233425_at	—	18q21.33
CNDP2	217752_s_at	CNDP dipeptidase 2 (metallopeptidase M20 family)	18q22.3
ZADH2	227049_at 227977_at 234977_at 1554239_s_at	zinc binding alcohol dehydrogenase domain containing 2	18q22.3
ADNP2	203322_at	ADNP homeobox 2	18q23
PRDM15	230777_s_at	PR domain containing 15	21q22.3
**Genes with lower DNA copy number and expression in metastatic OSCC**			
SSFA2	202506_at	sperm specific antigen 2	2q31.3
TPPP	230104_s_at	tubulin polymerization promoting protein	5p15.3
MAFK	226206_at	v-maf musculoaponeurotic fibrosarcoma oncogene homolog K (avian)	7p22.3
EIF3B	208688_x_at 203462_x_at	eukaryotic translation initiation factor 3, subunit B	7p22.3
FOXK1	226715_at	forkhead box K1	7p22.1
LAPTM4B	1554679_a_at	lysosomal protein transmembrane 4 beta	8q22.1
EIF2C2	225827_at	eukaryotic translation initiation factor 2C, 2	8q24
YAP1	213342_at	Yes-associated protein 1	11q13
PCSK7	203118_at	proprotein convertase subtilisin/kexin type 7	11q23-q24
DCXR	217973_at	dicarbonyl/L-xylulose reductase	17q25.3
CECR5	218592_s_at	cat eye syndrome chromosome region, candidate 5	22q11.1
HIRA	217427_s_at	HIR histone cell cycle regulation defective homolog A (S. cerevisiae)	22q11.21

### Survival analysis

**Figure 2:**
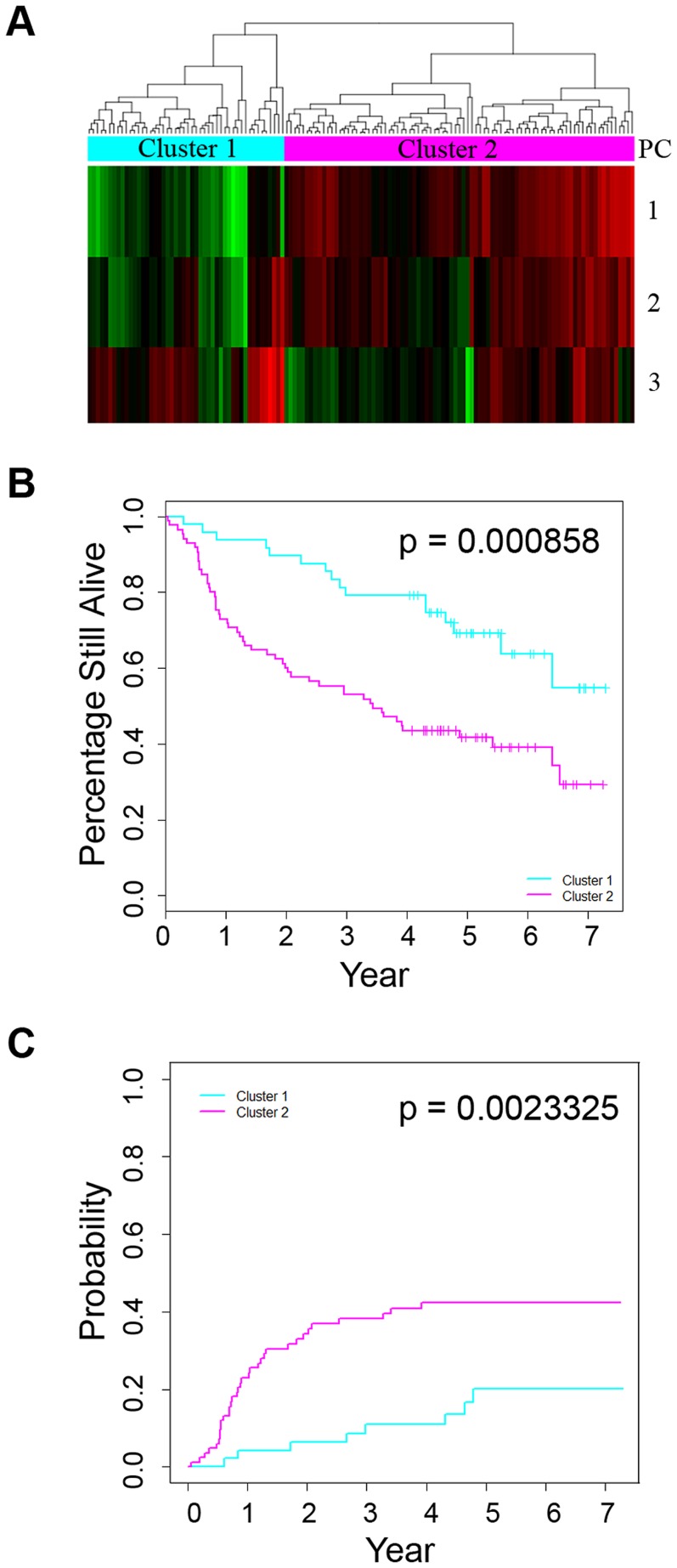
Association between DNA copy number–associated differentially expressed genes and survival. A) Hierarchical clustering of an independent dataset of 133 patients using the 95 CNA-associated differentially-expressed gene signature. Expression variances of the genes were summarized by principal components analysis. A hierarchical clustering analysis was performed using the first three principal component (PC) scores; B) Overall survival of the two patient clusters stratified by the 95-CNA-associated differentially expressed signature; C) Cumulative incidence of OSCC-specific death for the two patient clusters.

To test whether the patterns of CNA-associated genes that are differentially expressed can be used for OSCC prognosis, we evaluated the expression of the 95 transcripts in an independent dataset of 133 OSCC patients. Sixty five of the remaining 133 patients were alive at the end of the follow-up time (mean: 5.4 years; range: 4.0–7.3 years). The expression of the 95 transcripts in these 133 patients was first summarized using principal components analysis (PCA). A hierarchical clustering analysis using the first three principal component scores captured 99.6% of the variation in expression of the 95 transcripts and divided the 133 patients into two clusters; and the expression of the 95 transcripts in cluster 2 was consistent with the direction of the expression changes associated with metastatic OSCC (Figure 2A). Patients in the two clusters were similar in age, gender, and AJCC stage distribution (Table S3). A higher proportion of cluster 2 patients had T3/T4 primary tumors (45.9% vs. 25%) but cluster 1 patients were more likely to have nodal metastasis (62.5% vs. 50.6%). HPV16 was detected in 64.6% of patients in cluster 1 but in only 20% of patients in cluster 2. The 5-year overall survival for patients in cluster 2 was 41.7±5.45% (mean ± SE) compared to 69.1±6.98% for patients in cluster 1 (p = 0.000858, Figure 2B). The estimated cumulative mortality due to OSCC at 5 years for patients in cluster 2 was 40.9±5.46% compared to 18.78±6.19% for patients in cluster 1 (p = 0.0023325, Figure 2C). In multivariate analysis adjusting for age, sex, AJCC stage and HPV status, the overall and OSCC-specific mortality hazards ratios (HR) for patients in cluster 2 were 3.45 (95% CI: 1.84–6.50) and 4.75 (95% CI: 2.03–11.11), respectively (Table 2). To address potential residual confounding by the known association of HPV status with survival in oropharyngeal tumors [13], we repeated the analysis excluding HPV+ oropharyngeal tumors from the 133 patient cohort (n = 33). A hierarchical cluster analysis using the 95 gene signature yielded two main clusters of tumors and as above, the differential expression of the 95 transcripts in cluster 2 was consistent with that associated with metastatic OSCC (Figure S3A). Once again, the 5-year overall survival and OSCC cumulative mortality were significantly different between these two clusters (Figure S3B and S3C). In multivariate analysis adjusting for age, sex, AJCC stage and HPV status, the overall and OSCC-specific mortality hazards ratios (HR) for patients in cluster 2 were 2.65 (95% CI: 1.36–5.16) and 4.98 (95% CI: 1.74–14.21), respectively.

**Table 2:** Multivariate analysis for overall and OSCC–specific survival.

	Overall death		OSCC-specific death	
	HR (95% CI)*	*p-value*	HR (95% CI)*	*p-value*
Age	1.00 (0.98–1.02)	0.92	1.01 (0.98–1.04)	0.57
Gender (Male vs. Female)	0.87 (0.51–1.47)	0.60	0.74 (0.39–1.43)	0.38
HPV (high-risk vs. low-risk/negative)	1.44 (0.82–2.53)	0.20	1.99 (1.00–3.93)	0.05
Patients in cluster 2 vs. in cluster 1	3.45 (1.84–6.50)	1.22E-04	4.75 (2.03–11.11)	3.31E-04
AJCC (stage III/IV vs. stage I/II)	4.38 (2.15–8.93)	4.80E-05	5.91 (2.08–16.75)	8.38E-04

* HR: hazard ratios; CI: 95% confidence interval.

### Functional genomics to identify CNA–associated genes with the strongest impact on cell survival and growth

**Figure 3:**
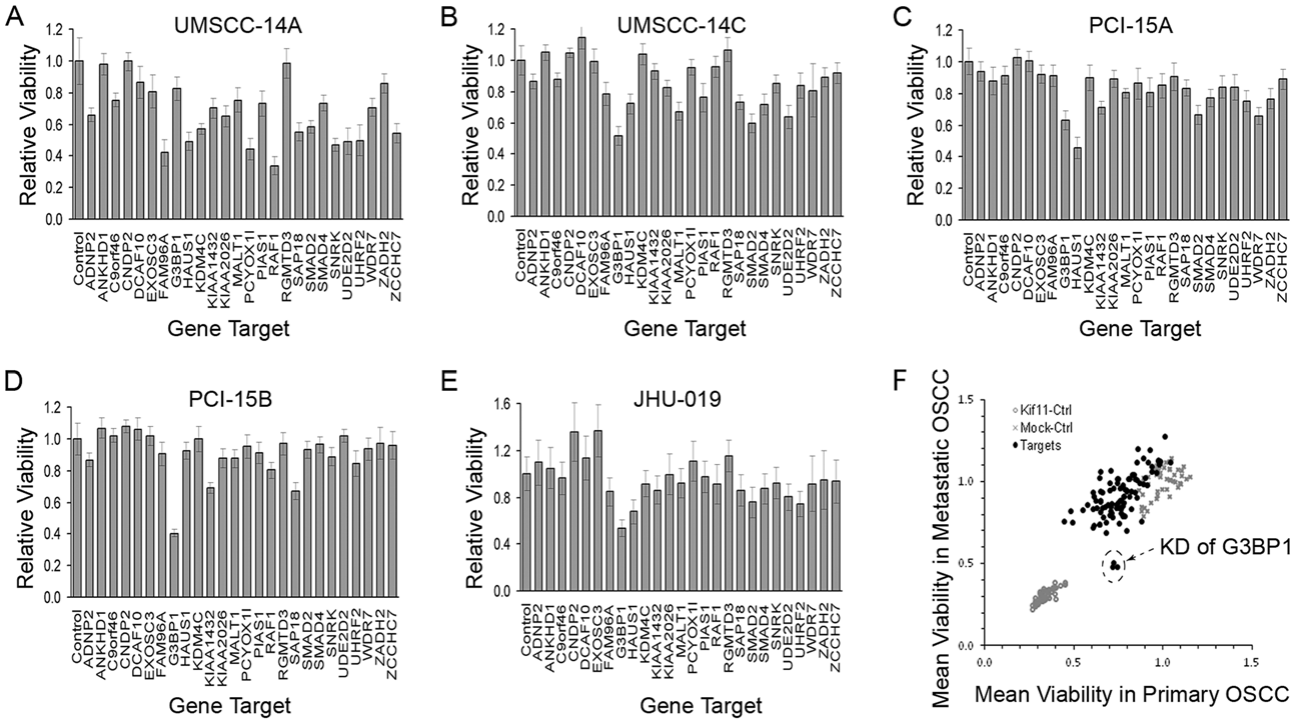
Results of siRNA knockdown screen. (A–E) Cell viability after the siRNA-mediated gene knock-down (KD) screen in UM-SCC-14A, UM-SCC-14C, PCI-15A, PCI-15B, and JHU-019. Cell viability was measured 4 days after the siRNA transfection. Results were presented as relative to control cells treated with transfection reagent only. Error bar represent the standard deviation of 9 repeats; (F) Average viability in cell lines derived from primary OSCC (x-axis) and from metastatic OSCC (y-axis). KD of Kiff11 and transfection reagent only was used as positive and negative control. Arrow indicates the three repeats of KD of G3BP1.

We first characterized the OSCC cell lines with respect to growth, migration rates and metastatic potential. OSCC cell line characteristics are shown in Table S4 and Figures S4 and S5. JHU-019 had the highest migration rate in the five OSCC cell lines. For the paired lines, the migration rate of cell lines derived from metastatic OSCC (i.e., UM-SCC-14C and PCI-15B) was higher in than those derived from primary tumor (i.e., UM-SCC-14A and PCI-15A) (Figure S4). In addition, all three cell lines tested in mouse xenografts by orthotopic injection into the tongue effectively produced squamous carcinoma at the sites of injection, as shown by both GFP fluorescence imaging and histological evaluation (data not shown). JHU-019 and PCI-15B demonstrated pronounced metastasis to cervical lymph nodes and had a shorter time to euthanasia due to tumor burden compared to PCI-15A, which did not show metastasis. This is consistent with PCI-15A being derived from a less aggressive, earlier-stage HNSCC, compared to the autologous recurrent, treatment-resistant tumor from which PCI-15B was established (data not shown) [14]. To identify CNA-associated genes unique to metastasis that are associated with tumor growth and that could be targeted against metastatic OSCC, we selected 26 out of the 95 transcripts with amplification and over-expression in metastatic OSCC to perform gene expression knockdown (KD) screens in the five established cell lines derived from early-stage primary OSCC (i.e., UM-SCC-14A and PCI-15A) and from late-stage OSCC (i.e., UM-SCC-14C, PCI-15B, and JHU-019 [14]). With ≥30% growth inhibition as a cutoff value, the KD of 18 of the 26 genes (69%) showed growth inhibition in at least one of five OSCC selected cell lines (Figure 3A–3E). We tested the KD of an additional gene subset from the human kinome in these cells, none of which were among the 95 genes in the signature. KD of only 149 out of 708 (21%) showed ≥30% growth inhibition in at least one of the cell lines, indicating that the 26 target genes from the 95 gene signature represent an enriched pool that is associated with OSCC cell survival (p = 3.421e-07, Figure S6). Although, on average, cell lines derived from early-stage, primary OSCC (i.e., UM-SCC-14A and PCI-15A) were more sensitive than cell lines derived from late-stage OSCC in the KD screen, late-stage OSCC (i.e., UM-SCC-14C, PCI-15A, and JHU-019) were more sensitive to the KD of GAP SH3 Binding Protein 1 (G3BP1) (Figure 3F).

### Confirmation of G3BP1 KD effects on cell survival

**Figure 4:**
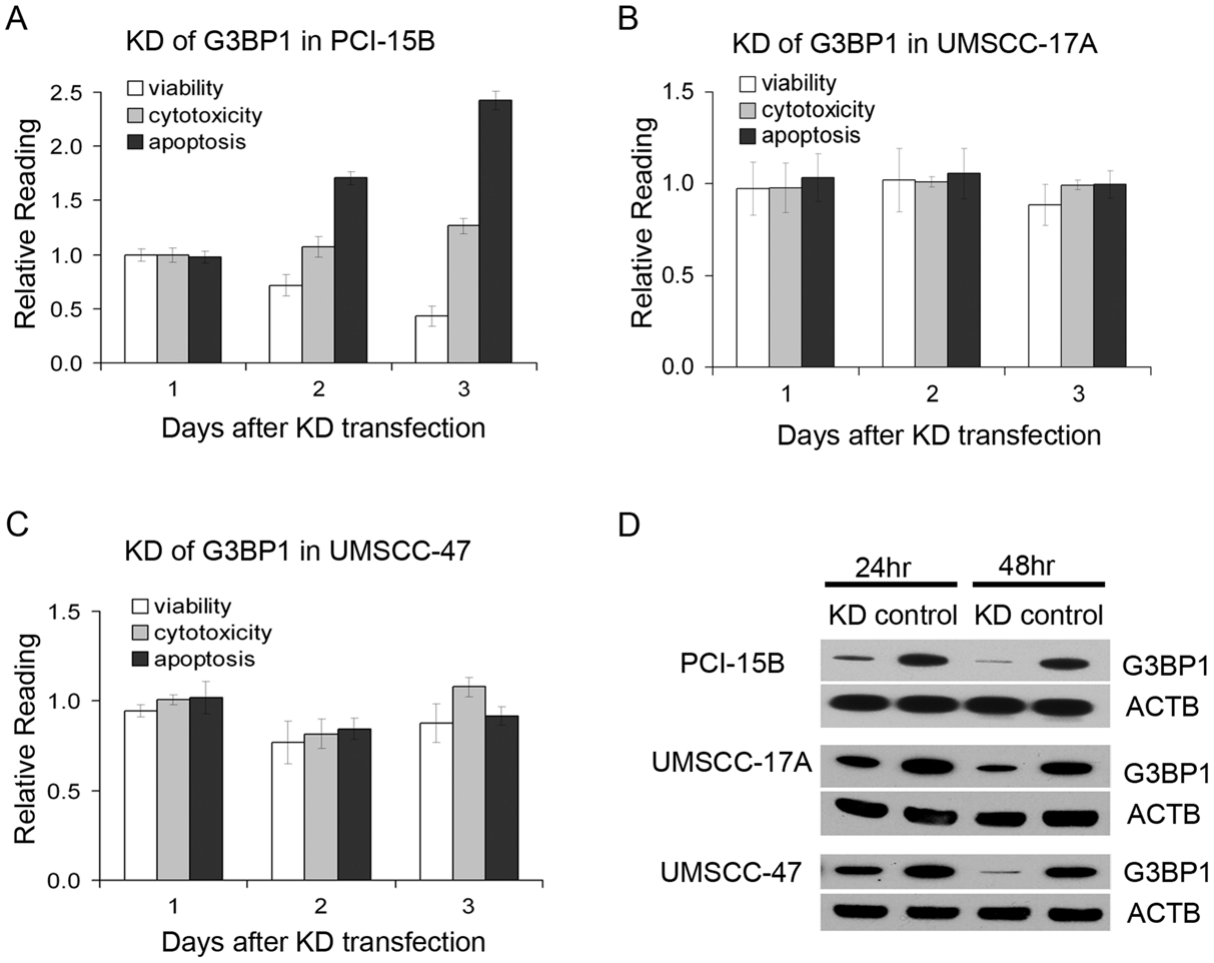
Effects of G3BP1 knockdown in OSCC. Cell viability, cytotoxicity, and apoptosis in OSCC cell lines PCI-15B (A), UM-SCC-17A (B), UM-SCC-47 (C) after the KD of G3BP1. The results are presented as relative to the reading in control wells that are transfected with negative control siRNA. Error bar indicates standard deviation of the readings in three repeat wells; (D) Confirmation of the KD of G3BP1 in these cell lines with western blot. Beta-actin (ACTB) is used as a loading control.

To confirm the effects of G3BP1 siRNA knockdown in OSCC cells, we first measured G3BP1 mRNA level with real-time quantitative RT-PCR after KD transfection of each of the three pooled siRNAs used in the high-throughput screen, and this identified the siRNA that knocked down the G3BP1 most effectively (data not shown). We knocked down G3BP1 using this siRNA in PCI-15B and observed a decrease in cell viability and an increase of cell apoptosis that accompanied the G3BP1 KD, but there were no significant changes in cytotoxicity in PCI-15B (Figure 4A). G3BP1 knockdown also decreased cell viability and promoted cell apoptosis in the two other cell lines, UM-SCC-14C and JHU-019 (data not shown). As all these three tested lines had disruptive mutations in *TP53* (as determined by Poeta et al [15], data not shown), we also tested the KD effects on two p53 wild type OSCC cell lines derived from a primary OSCC (UM-SCC-17A) and from a metastatic disease (UM-SCC-47), respectively [14]. However, there were no significant changes in viability, cytotoxicity or apoptosis when G3BP1 was knocked down in these cell lines (Figure 4B–4C). Given that G3BP1 was previously reported as a Ras-GTPase-activating protein (RasGAP) SH3-domain-binding protein that enabled the *Ras* pathway, we explored whether G3BP1 KD influenced the *Ras* pathway in the OSCC cancer cells. We measured both the activated forms of Ras and p44/42 MAPK (Erk1/2) in PCI-15B cells following G3BP1 KD siRNA transfection. No significant changes were detected in either of the two proteins (Figure S7).

## Discussion

To understand the importance of DNA copy number aberrations in OSCC progression, we sought to identify DNA copy number aberrations unique to carcinoma cells found in metastatic lymph nodes that are associated with changes in the expression of encoded genes. Moreover, we reasoned that since metastatic tumor cells have de-regulated apoptotic pathways [16], we could leverage on this survival advantage to find those genes associated with the growth-promoting pathways inherent to these cells. Using a large-scale siRNA interference screen, we further prioritized functionally relevant amplified/over-expressed growth-promoting genes associated with OSCC metastasis. We showed that CNAs-associated transcripts differentially expressed in carcinoma cells with an aggressive phenotype (i.e., metastatic to lymph nodes) can be biomarkers with both prognostic information and functional relevance. In particular, we demonstrated the potential of G3BP1 as a novel therapeutic target in metastatic OSCC.

DNA copy number profiling of the 37 samples in the training cohort detected most of the previously reported CNA associated with poor outcome in patients with OSCC or head and neck squamous cell carcinoma [17]. Interestingly, we detected more genomic alterations in carcinoma cells from non-metastatic primary OSCC than in those from metastatic OSCC, which is consistent with previous findings in other cancer types [18], [19]. It may be an indication that not all genetic alterations are related to metastasis, thus supporting the theory that metastasis is derived from a subclone in the primary tumor. The DNA copy number in the four chromosomal regions (3p25.3-21.1, 5q31-35.3, 9p24.3- 22.3, and 18q21-23), which harbor 59 of the 95 transcripts, is significantly higher in metastatic OSCC than in non-metastatic primary OSCC. Subsequent KD test showed that multiple genes involved in the survival of OSCC cell lines locate in these regions. Thus multiple gene expression changes that may be essential to OSCC cancer progression may be the result of the same CNA event in these regions.

Accumulating evidence supports the hypothesis that metastasis derives from a subclone of cancer cells in a late-stage primary tumor [20], [21]. In this study, we focused on metastasis by comparing carcinoma cells from non-metastatic primary carcinomas with carcinoma cells metastatic to lymph nodes, rather than a pair-wise comparison between carcinoma cells from primary OSCC and corresponding metastatic carcinoma cells isolated from lymph nodes. This distinction is important, because there is evidence that prior to metastasis, the carcinoma in the primary site already contains malignant cells with genetic changes associated with metastatic potential, as we and others have shown [22]–[27]. Given that the comparison of genetic changes between carcinoma cells in lymph nodes vs. those in the primary site of non-metastatic carcinomas involves two distinct micro-environmental milieus, we used laser-capture microdissection to enrich for carcinoma cells. Because of this, we could not identify genes in the cancer-associated stroma that may be essential to the metastasis process. In addition, due to the relatively small sample size of the training set, the genetic variances among individual patients may reduce the power to identify true differences in CNAs or gene expression dysregulation between these two groups of tumor cells.

The 95 gene signature was strongly associated with OSCC survival in an independent patient cohort. We focused on survival as an outcome because understanding that metastasis is a strong clinical predictor of survival, we reasoned that those genomic aberrations, and associated changes in gene expression, unique to tumor cells from metastatic lymph nodes would likely be candidate biomarkers of patient outcomes. It is possible that the 95 gene signature associated with poor prognosis represents a correlative phenomenon of cell survival, rather than being causative of the metastatic process per se, which is complex and involves a number of phenotypes such as enhanced survival, migration, proliferation, anoikis, etc. In fact, one motivation for our study design was to identify genes associated with aggressive tumor, which is associated with these phenotypic attributes, and to identify genes that are involved in conferring cells a survival advantage (regardless of the mechanisms through which survival is affected).

It is noteworthy that our 95 gene set was not associated with differences in nodal metastasis in the independent patient cohort. There are multiple reasons for this finding. First, the types of tissue analyzed were different between the training and validation cohorts (i.e., the training set consisted of tumor tissue from lymph nodes and non-metastatic primaries whereas the validation set consisted of biopsy samples derived from the primary site only). Secondly, the training set consisted of tumor cells only whereas the validation set represented a mixture of tumor and stromal cells. Thirdly, we may have lacked the power to detect statistically significant differences in nodal status. Fourth, the primary sites of metastatic tumors are likely composed of mixtures of clonal populations – some with metastatic potential and some without. In contrast, we believe that tumor cells in metastatic lymph nodes represent a more homogeneous clonal pool, at least with respect to the metastatic phenotype.

Our finding that 18 of the 26 gene targets tested in the KD screen showed an effect on the viability of OSCC cells indicates that this strategy, if validated in larger studies, could prove successful in identifying biomarkers of OSCC metastasis with therapeutic potential. It is possible that with the criteria chosen for siRNA validation (growth suppression >30% in at least one cell line), other genes unrelated to OSCC cell survival might also meet this threshold. However, the effects on OSCC cell survival upon siRNA KD of an additional 708 human kinases showed that our 26 target genes had a significantly higher proportion of genes involved in OSCC cell survival. We selected genes from the human kinome for this comparison because kinases are known to regulate signaling pathways which could affect growth and survival. Thus, this represented a comparable pool that could also be enriched with genes relevant for the same phenotype for which the 26 target genes were being tested. Our results suggest that our approach was successful at identifying an enriched gene set associated with OSCC progression.

In this study, we focused on G3BP1, because among the 18 genes whose KD induced growth suppression, G3BP1 KD suppressed growth in 4 of the 5 cell lines, and, moreover, the effect was stronger in the lines derived from late-stage OSCC. This feature, if validated further, could prove attractive in the search for targeted therapies against head and neck carcinomas. G3BP1 was first identified as a Ras-GTPase-activating protein (GAP) binding protein as it co-immunoprecipited with GAP [28]. Over-expression of G3BP1 has been detected in a number of human cancers including breast, lung, prostate, colon, thyroid, esophageal, and head and neck cancers [29], and over-expression has also been associated with lymph node metastasis and worse survival in esophageal squamous cell carcinoma [30]. G3BP1 has been implicated in several cancer-associated survival pathways including *Ras* [28], [31]. Recently however, a study by Annibaldi et al. put into question the interaction between G3BP1 and RasGAP [32]. The fact that G3BP1 KD did not affect ERK1/2 phosphorylation status or the active form of Ras protein in our cell lines implies that growth suppression and apoptosis was not through inhibition of this pathway. G3BP1 also exhibits cell-cycle dependent RNase activity that affects stability of *c-myc* and *Tau* mRNA. G3BP1 has been shown to participate in stress granule (SG) assembly, and the stress granule is the site where some mRNAs are stored during stress conditions and where the ultimate fate of these mRNAs is decided once stress has subsided [33], [34]. Formation of SGs in cells may inhibit apoptosis Arimoto K [35], and it is possible that this is one mechanism by which G3BP1 inhibition induced apoptosis in our cell lines.

Another question to address is why induction of apoptosis seemed to be more pronounced in cell lines derived from late-stage OSCC after G3PB1 KD. Since G3BP1 KD induced apoptosis preferentially in the metastatic cell lines with disruptive mutations in *TP53* gene, it is possible that this effect might be p53-dependent. Loss of *TP53* function is one of the most common genetic alterations in squamous cell carcinoma (SCC) of the head and neck [15], [36], [37] and head and neck tumors with disruptive *TP53* mutations have been found to be more likely metastatic and to have worse survival [15]. This latter point is relevant to this study, in that oncogenesis-mediated proliferation has been shown to be p53 pathway-dependent [38]. Indeed, a recent study implicated G3BP1 in the p53 pathway, as KD of G3BP1 led to up-regulation of p53 levels and activity in breast cancer cell line MCF7 [29]. In lung cancer cell line H1299, KD of G3BP1 suppressed anchorage-independent cell growth [39]. Thus, our results regarding the effects of G3BP1 inhibition appear consistent with those found in other studies. However, given the diversity of functions with which G3BP1 has been associated, further studies are warranted to elucidate the mechanism of action.

In conclusion, we have identified a 95-gene set based on CNA-associated gene dysregulation unique to OSCC metastasis which is prognostic in an independent dataset. In addition, we identified 18 amplified/over-expressed genes which appear to promote cell viability and growth. In particular, we identified G3BP1 as a potential target against metastatic OSCC. Further investigation is warranted to examine the biologic role of these CNA-associated differentially expressed genes and to determine the mechanism by which G3BP1 inhibition induces apoptosis in metastatic OSCC cells.

## Materials and Methods

### Tissue collection, OSCC genome, and transcriptome profiling

OSCC tissue specimens were collected from consented patients as previously described [40]. All research involving human participants have been approved by our institutional review board. Informed consents have been obtained from our research participants. In addition to the 20 samples of lymph node with metastatic OSCC previously reported [41], we collected an additional 17 stage I/II node-negative (*non-metastatic*) primary tumors. Genome and transcriptome profiling was performed as previously described with minor changes [41]. Briefly, tissue specimens were embedded in Tissue-Tek OCT compound (Sakura Fineteck USA, Torrance, CA), sectioned, and stained with Hematoxylin and Eosin (H&E). Tumor cells were outlined by our study pathologist and collected using a Veritas laser capture microdissection system (Life Technologies, Carlsbad, CA). DNA and RNA were isolated from the same OSCC cell population using AllPrep DNA/RNA Micro Kit (Qiagen, Valencia, CA) as previously described [42]. The quantity and quality of the purified DNA and RNA were assessed with the ND-1000 spectrophotometer (Thermo Fisher Scientific, Rockford, IL), and the integrity of the RNA was verified using the Agilent 2100 bioanalyzer (Agilent Technologies, Santa Clara, CA). For genome profiling, 250 ng of genomic DNA was amplified, labeled and hybridized onto Human Mapping 250K *Nsp* I single-nucleotide polymorphism (SNP) array (Affymetrix, Santa Clara, CA) following the manufacturer's instructions. For transcriptome profiling, 40 ng of purified total RNA was amplified using RiboAmp Plus 1.5-round kit (Life Technologies), biotin-labeled and fragmented using GeneChip IVT Labeling kit and hybridized to the Human Genome U133 Plus 2.0 array (Affymetrix). Intensity of the hybridized features on the 250K Nsp I SNP and U133 Plus 2.0 arrays was acquired with GCOS (v1.4) software (Affymetrix). All genome and transcriptome profiles passed the manufacturer's QC standards.

### Identification of CNA–associated differentially expressed genes in metastatic OSCC

Genes with differential expression between non-metastatic primary and metastatic OSCC were identified using linear regression, as previously described [41]. Briefly, transcriptome profiles were normalized using the R package gcRMA (www.bioconductor.org). Probe sets that either showed no variation across the samples being compared or that were expressed at very low magnitude were eliminated, to reduce the multiple statistical testing penalty. Expression values were analyzed using a regression-based estimating equations approach implemented in GenePlus software [43], [44]. The integrative analysis of DNA copy number and gene expression was performed as previously described with minor modifications [41]. Briefly, SNP genotyping was determined by BRLMM algorithm in Genotyping Console (v3.0) software (Affymetrix). The SNP array results were normalized in dChip (http://biosun1.harvard.edu/~cli/complab/dchip/). DNA copy number was estimated using the R package cghFlasso (www.bioconductor.org) in reference to the Affymetrix 48 HapMap normal samples. The location of each transcript with differential expression between non-metastatic primary and metastatic OSCC was mapped to the human genome (Build 36.1) using Affymetrix's annotation file ‘HG-U133_Plus_2.na27’. SNPs within 250 kb upstream and downstream of each transcript were identified using Affymetrix's annotation file ‘Mapping250K_Nsp.na26.annot.csv’. Correlation between CNA and gene expression across all OSCC samples were assessed using a robust regression model. CNA differences between non-metastatic primary OSCC and lymph node metastases were assessed with Wilcoxon Rank-Sum Test.

### Survival analysis of CNA–associated differentially expressed genes

We used a previously reported independent dataset with expression profiles from 167 OSCC patients (NCBI GEO database accession # GSE30784 [40]) to determine whether an expression signature of the CNA-associated genes unique to metastasis would cluster patients with more aggressive tumors and, subsequently, worse survival. Of the 167 patients, we eliminated 34 patients who were among the patients used in this study to identify CNA-associated genes unique to metastasis. Human papillomavirus (HPV) status was determined as previously described in Lohavanichbutr et al [45]. Gene expression values of the remaining 133 patient-testing dataset were normalized using the R package gcRMA. The expression values of the CNA-associated differentially expressed genes were first summarized in a principal components analysis (PCA). A hierarchical clustering analysis was then performed using the first 3 PCA scores. The clinical characteristics among the two subgroups of clustered patients were compared by using Fisher's exact test. Follow-up time for analyses of overall survival was calculated from the date of surgery to the date of death, loss-to-follow-up, or last date of contact, whichever came first, according to the Kaplan-Meier method. Differences between groups were assessed with the log-rank test. We did not compute OSCC-specific Kaplan Meier survival estimates because of possible informative censoring due to death from other causes. Rather, we estimated OSCC-specific cumulative mortality using R package “cmprsk” [46]–[48], which account for competing risk events. Hazard ratios (HR) for overall survival were calculated with multivariable Cox regression analysis adjusting for gender, age, HPV status, and AJCC stage. To eliminate potential confounding of the association between the 95 gene signature and survival by HPV presence in the oropharyngeal carcinomas, the expression values of the CNA-associated differentially expressed genes were summarized in a PCA excluding the HPV+ oropharyngeal cases from the 133 patient cohort, and the same subsequent analyses were then performed as outlined above.

### Characterization of OSCC cell lines

To test whether knockdown of the CNA-associated differentially expressed genes would affect OSCC cell viability, we used the following OSCC cell lines: UM-SCC-14A/14C and PCI-15A/15B both autologous pairs derived from patients presenting with early stage OSCC and then with subsequent recurrent tumor, and cell line JHU-019 derived from a late-stage OSCC patient [14], [49]. To characterize these cell lines with respect to their metastatic potential and survival, we assessed the cell growth (including anchorage-independent growth) and migration rates *in vitro*, and formation of cervical nodal metastasis in an orthotopic xenograft mouse model Henson B [50]. Anchorage-independent and dependent cell growth rates were established by seeding cells at 2000cells/well into Costar 96-well white clear-bottom tissue culture plates coated with and without Poly-HEMA in 200 µl of culture medium (DMEM for UM-SCC and PCI cell lines, and RPMI 1640 for JHU-019) containing 10% fetal bovine serum (FBS). For Poly-HEMA coating, the plates were incubated with Poly-HEMA (Sigma-Aldrich Corp., St. Louis, MO. cat # P3932) at 5 ug/mL in 95% ETOH at 80°C for 1 hour to evaporate the ethanol and then sterilized by UV irradiation (120 joules/cm^2^, 10 min) in a Spectrolinker UV Crosslinkers (Spectronics, Westbury, NY). Viable cells in each well were measured with the CellTiter-Glo Luminescent Cell Viability Assay kit (Promega, Madison, WI). Cell migration rates were determined with wound-healing assay. Briefly, cells were plated into 6-well plates at a density of 1×10^6^/well. After attachment, the cells were starved in medium with 0.1% serum overnight, and a sterile 200 µl pipette tip was used to scratch the cells to form a wound. The cells were then washed twice with 1×PBS and maintained in medium with 0.1% FBS. Pictures were taken at the 0, 8 and 20 hrs. Given the fast rate of wound closure in the JHU-019 line, pictures were taken at 0, 3, 6 and 12 hrs. Cell migration rates were calculated with ImageJ (http://rsbweb.nih.gov/ij/). The ability to form cervical nodal metastasis in orthotopic xenograft mouse model was assessed by injection of 5000 GFP-labeled cells in 50% Matrigel (BD Biosciences, Sparks, MD) into the tongues of NOD/SCID mice. Lymph node metastases were detected using a Xenogen IVIS-200 Optical In Vivo Imaging System (Caliper Life Sciences, Hopkinton, MA) and confirmed histopathologically. Animal experiments and procedures were conducted following approval in accordance with the Institutional Animal Care and Use Committee at Fred Hutchinson Cancer Research Center.

### siRNA–mediated gene expression knockdown

To select CNA-associated, differentially expressed genes in OSCC metastasis for a siRNA-knockdown, we first screened on the basis of the following two criteria: 1) probe sets/transcripts had to represent known genes; and 2) the genes had to be amplified in at least two metastatic OSCC specimens. After filtering out genes using the first two criteria, we superimposed biologic domain knowledge using Ingenuity Pathway Analysis, and we prioritized gene candidates associated with pathways or molecular functions relevant in cancer. Transfection optimization was performed for a panel of established OSCC cell lines, JHU-019, UM-SCC14A and C, and PCI-15A and B. First, cells were seeded in 384-well tissue culture plates using a Matrix WellMate machine (Thermo Fisher Scientific, Rockford, IL). Twenty-four hours after cell seeding, cells were transfected with either a mixture of siRNAs against KIF11 (which arrests cells in mitosis) or control siRNAs (mock vs. non-targeting universal siRNA) along with transfection reagent, using a CyBio Vario 384/25 automated liquid handling workstation (CyBio AG, Germany). Cell viability was determined using CellTiter-Glo luminescent cell viability assay kit (Promega, Madison, WI) on an EnVision Multilabel reader (PerkinElmer, San Jose, CA). The goal for optimization was potent killing with KIF11 at the lowest toxicity possible in both the mock and universal controls. Tested conditions included: 1) transfection reagents: Lipofectamine RNAiMAX (Life Technologies) and DharmaFECT 1 (Thermo Fisher Scientific); 2) cell seeding densities: 500, 1000, 2000 cells per well; 3) siRNA concentrations: 2.5–50 nM final concentration; 4) transfection reagent concentrations: 0.01–0.06 µl per well; and 5) follow-up times: 72 hr and 96 hr after siRNA transfection. The optimal condition for the siRNA screen in the five OSCC cell lines was determined to be a seeding density of 500 cells in 50 µl of medium to each well on the 384-well plate 24 hr before transfection. siRNA (final concentration: 25 nM) and RNAiMAX (0.05 µl) was used in each well for gene knockdown. Cell viability was measured 96 hr after transfection. Once transfection conditions were optimized, knockdown experiments against targets of interests were performed using a pool of three siRNAs per target designed and manufactured by Qiagen (see siRNA sequence information in Table S5). In addition, we used the MISSION siRNA Human Gene Family Set kinase panel to knock down the expression of an additional 708 genes (713 minus 5 human kinases which were among the 95 genes in our signature) (Sigma-Aldrich Corp, see siRNA sequence information in Table S6). The viability of these cells was measured 4 days after the siRNA-mediated gene knockdown transfection using the CellTiter-Glo luminescent cell viability assay kit. Growth suppression was defined as ≥30% loss in cell viability in at least one cell line. Boxplots were used to display the effects of siRNA knockdown (i.e., no growth suppression vs. growth suppression) for each of the 26 vs. 708 targeted genes sets. Fisher's exact test was used to test differences in the KD status between the 26 vs. the 708 gene sets.

Subsequent knockdown experiments for the G3BP1 gene followed the same conditions as above (with scaling up) using siRNA target sequence 2 (see sequence information in Table S5). AllStars Negative Control siRNA (Qiagen) was used as a non-silencing siRNA negative control. Cell viability, cytotoxicity, and apoptosis were measured with ApoTox-Glo Triplex Assay (Promega, Madison, WI).

### Quantitative real-time PCR (qPCR)

Total RNA was extracted using RNeasy Mini Kit (Qiagen). Five micrograms of RNA was converted into cDNA with SuperScript III First-Strand Synthesis SuperMix kit (Life Technologies). Gene expression was determined with Platinum SYBR Green qPCR SuperMix-UDG w/ROX (Life Technologies) on an AB 7900HT machine (Life Technologies). Primer pairs used in qPCR were: AGTTGCGTGAGGGGTTTGTA and GGGGAAAAGAGTCAAATATGTCC (for measuring G3BP1 variant 1), AGTTGCGTGAGGGGTTTGTA and GGGGACTAGGCTTCTCCATC (for measuring G3BP1 variant 1 and variant 2), CGACCACTTTGTCAAGCTCA and TTACTCCTTGGAGGCCATGT (for measuring GAPDH).

### Western blot and Ras activity assay

To validate siRNA knock-down of candidate genes at the protein level and to determine the effect on downstream signal transduction, we performed Western blot analysis for GTPase activating protein (SH3 domain) binding protein 1 (G3BP1), the selected target from the siRNA screen described above. Cells were lysed using M-PER Mammalian Protein Extraction Reagent supplemented with Halt Protease and Phosphatase Inhibitor Cocktail (Thermo Fisher Scientific). Extracted proteins were quantified by a BCA protein assay (Thermo Fisher Scientific). Fifteen micrograms of each protein specimen was revealed on a NuPAGE 4–12% Bis-Tris mini gel (Life Technologies) and transferred onto an Immobilon-P PVDF membrane (Millipore, Billerica, MA). For Ras activity assay, phosphorylated Ras was pulled down from cell lysates using the Ras Activation Assay Kit (Millipore, Billerica, MA) following the manufacturer's protocol. Total Ras and phosphorylated Ras were detected using the anti-Ras mouse monoclonal clone RAS10 included in the kit. Antibodies used for detecting other proteins were mouse monoclonal anti-G3BP (BD Biosciences, San Jose, CA); rabbit monoclonal anti-p44/42 MAPK (Erk1/2) and anti-phospho-p44/42 MAPK (Erk1/2) (Cell Signaling, Danvers, MA); and rabbit polyclonal anti-ACTB (ProteinTech, Chicago, IL). The secondary antibodies used were ZyMax horseradish peroxidase (HRP) conjugated goat-anti-mouse or goat-anti-rabbit IgG (Life Technologies). HRP was detected with SuperSignal West Pico Chemiluminescent Substrate kit (Thermo Fisher Scientific).

## Supporting Information

Figure S1 Consensus plot of genome-wide copy number gains and losses in tumor cells from non-metastatic primaries (A) and metastatic lymph nodes (B). Colored vertical lines indicate percentages of copy number gains (red)/losses (green) among the chromosomes (CNA was defined as the cancer-normal DNA copy number ratio either <0.93 or >1.07). Arrows in (B) indicate the location of CNA-associated differentially expressed genes in metastatic OSCC.
(PPTX)

Figure S2 Summary of SNP probes copy number status in tumor cells from non-metastatic primaries and metastatic lymph nodes. Shown are histograms showing gains (red, top panel) and losses (green, bottom panel) in the 17 non-metastatic primary OSCC (left panel) and the 20 nodal metastases (right panel).
(PPTX)

Figure S3 Association between DNA copy number-associated differentially expressed genes and survival in the validation dataset excluding HPV+ oropharyngeal carcinomas. A) Hierarchical clustering of the 133-patient dataset excluding the HPV+ oropharyngeal carcinomas using the 95 CNA-associated differentially-expressed gene signature. Expression variances of the genes were summarized by principal components analysis. A hierarchical clustering analysis was performed using the first three principal component (PC) scores; B) Overall survival of the two patient clusters stratified by the 95-CNA-associated differentially expressed signature; C) Cumulative incidence of OSCC-specific death for the two patient clusters.
(PPTX)

Figure S4 Characterization of cell migration capability of OSCC cell lines by wound-healing assay. Cells were plated into 6-well plates at a density of 1×10^6^/well. A sterile 200 µl pipette tip was used to scratch the cells to form a wound. The cells were then washed twice with 1×PBS and maintained in medium with 0.1% FBS. Pictures were taken at the 0, 8 and 20 hrs. Given the fast rate of wound closure in the JHU-019-SCC line, pictures were taken at 0, 3, 6 and 12 hrs. (A) Representative images show cell migration (wound-healing) at 0, 8, and 20 hr. time points for UM-SCC14A and 14C lines. (B) Cell migration rate was measured using ImageJ software (Wayne Rasband, NIH, Bethesda, MD). Results are present as the average percentage of wound-healing rate from 3 wounds. Error bars represent the standard deviation.
(PPTX)

Figure S5 Anchorage dependent and independent growth rates of OSCC cell lines. Growth rates with and without anchorage dependence were determined using standard 96-well tissue culture plates (A) and the plates coated with Poly-HEMA (B). The growth rates were measured with CellTiter-Glo Luminescent Cell Viability Assay kit (Promega, Madison, WI). The growth rates are presented as the average percentage of growth relative to day 0 from 3 repeated wells and error bars represent the standard deviation.
(PPTX)

Figure S6 Boxplots showing growth suppression effects of siRNA knockdown experiments targeting the 26 vs. the 708 gene set. Growth suppression was defined as ≥30% loss in cell viability in at least one cell line and labeled as “Minimum Survival Rate” in the y-axis. The boxplots show the median and the first and third quartile range (IQR) of the Minimun Survival Rate. The whiskers show the minimum and maximum values that are within 1.5× the IQR, and the dots beyond the whisker represent the outliers defined as values beyond Q3+1.5XIQR or Q1-1.5XIQR.
(PPTX)

Figure S7 G3BP1 knockdown and Ras activity. The protein levels of G3BP1, phosphorylated and total Ras, phosphorylated and total Erk1, 2 in PCI-15B are shown at 48 hr and 72 hr after G3BP1 KD. Beta-actin is used as a loading control.
(PPTX)

Table S1 Characteristics of patients with metastatic and non-metastatic OSCC.
(XLSX)

Table S2 Ingenuity Pathway Analysis top functional categories of the CNA-associated differentially expressed genes.
(PPTX)

Table S3 Characteristics of OSCC patients in cluster 1 and cluster 2.
(XLSX)

Table S4 Characteristics of the OSCC cell lines used for siRNA functional screens.
(PPTX)

Table S5 siRNA sequences used for gene knockdown.
(XLSX)

Table S6 MISSION siRNA Human Gene Family Set used for kinase knockdown.
(XLSX)
