# Supplementary material for: Integrative Genomics in Combination with RNA Interference Identifies Prognostic and Functionally Relevant Gene Targets for Oral Squamous Cell Carcinoma
Source: PLoS Genet. 2013 Jan 17;9(1):e1003169. doi: 10.1371/journal.pgen.1003169 (PMC3547824; doi:10.1371/journal.pgen.1003169)
Supplement: Figure S1 — Consensus plot of genome-wide copy number gains and losses in tumor cells from non-metastatic primaries (A) and metastatic lymph nodes (B). Colored vertical lines indicate percentages of copy number gains (red)/losses (green) among the chromosomes (CNA was defined as the cancer-normal DNA copy number ratio either <0.93 or >1.07). Arrows in (B) indicate the location of CNA-associated differentially expressed genes in metastatic OSCC. (PPTX) [file pgen.1003169.s001.pptx]

## Slide 1
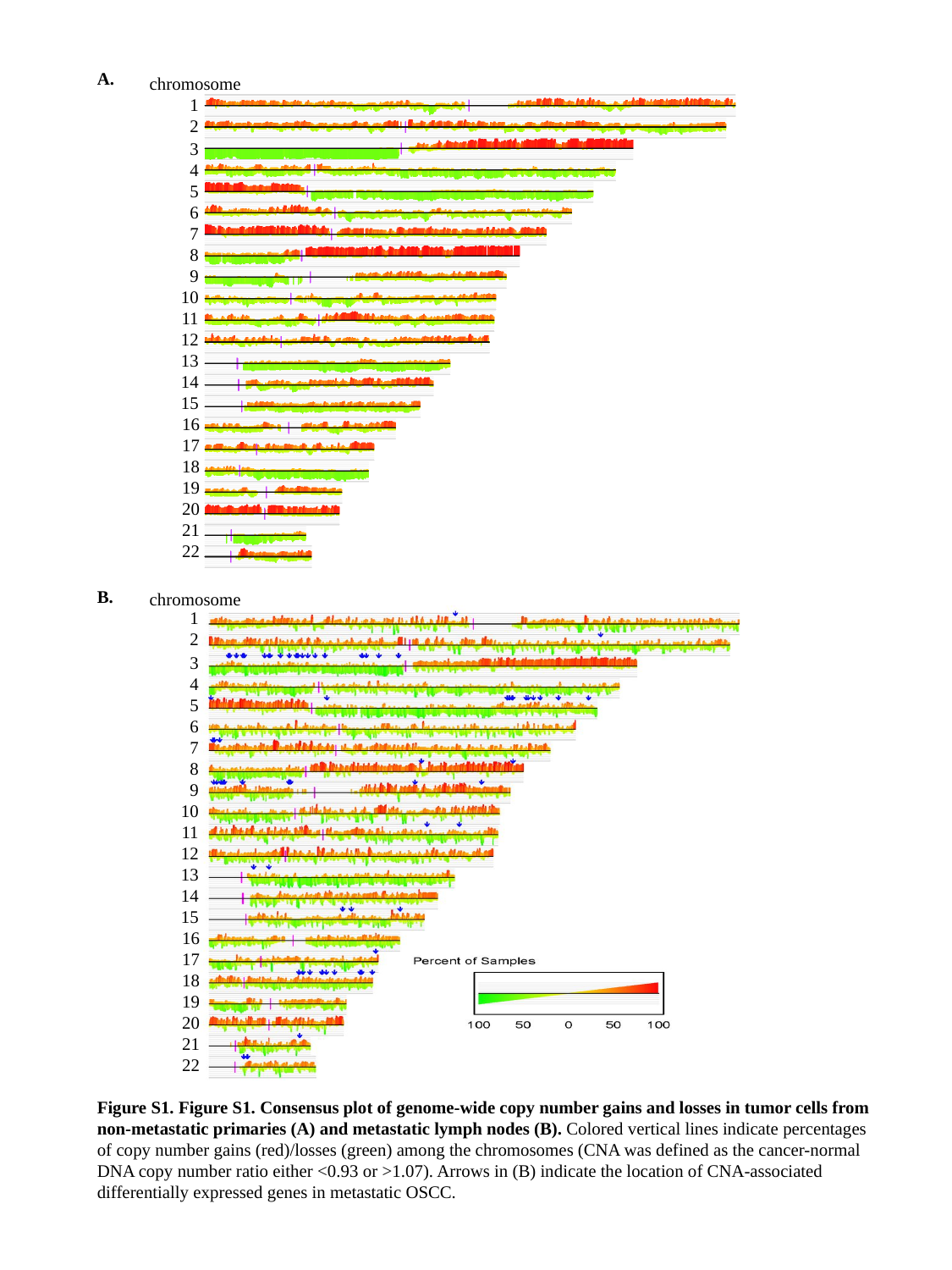

A.
chromosome
1
2
3
4
5
6
7
8
9
10
11
12
13
14
15
16
17
18
19
20
21
22
B.
chromosome
1
2
3
4
5
6
7
8
9
10
11
12
13
14
15
16
17
18
19
20
21
22
Figure S1. Figure S1. Consensus plot of genome-wide copy number gains and losses in tumor cells from non-metastatic primaries (A) and metastatic lymph nodes (B). Colored vertical lines indicate percentages of copy number gains (red)/losses (green) among the chromosomes (CNA was defined as the cancer-normal DNA copy number ratio either <0.93 or >1.07). Arrows in (B) indicate the location of CNA-associated differentially expressed genes in metastatic OSCC.
