# Supplementary material for: Integrative Genomics in Combination with RNA Interference Identifies Prognostic and Functionally Relevant Gene Targets for Oral Squamous Cell Carcinoma
Source: PLoS Genet. 2013 Jan 17;9(1):e1003169. doi: 10.1371/journal.pgen.1003169 (PMC3547824; doi:10.1371/journal.pgen.1003169)
Supplement: Figure S2 — Summary of SNP probes copy number status in tumor cells from non-metastatic primaries and metastatic lymph nodes. Shown are histograms showing gains (red, top panel) and losses (green, bottom panel) in the 17 non-metastatic primary OSCC (left panel) and the 20 nodal metastases (right panel). (PPTX) [file pgen.1003169.s002.pptx]

## Slide 1
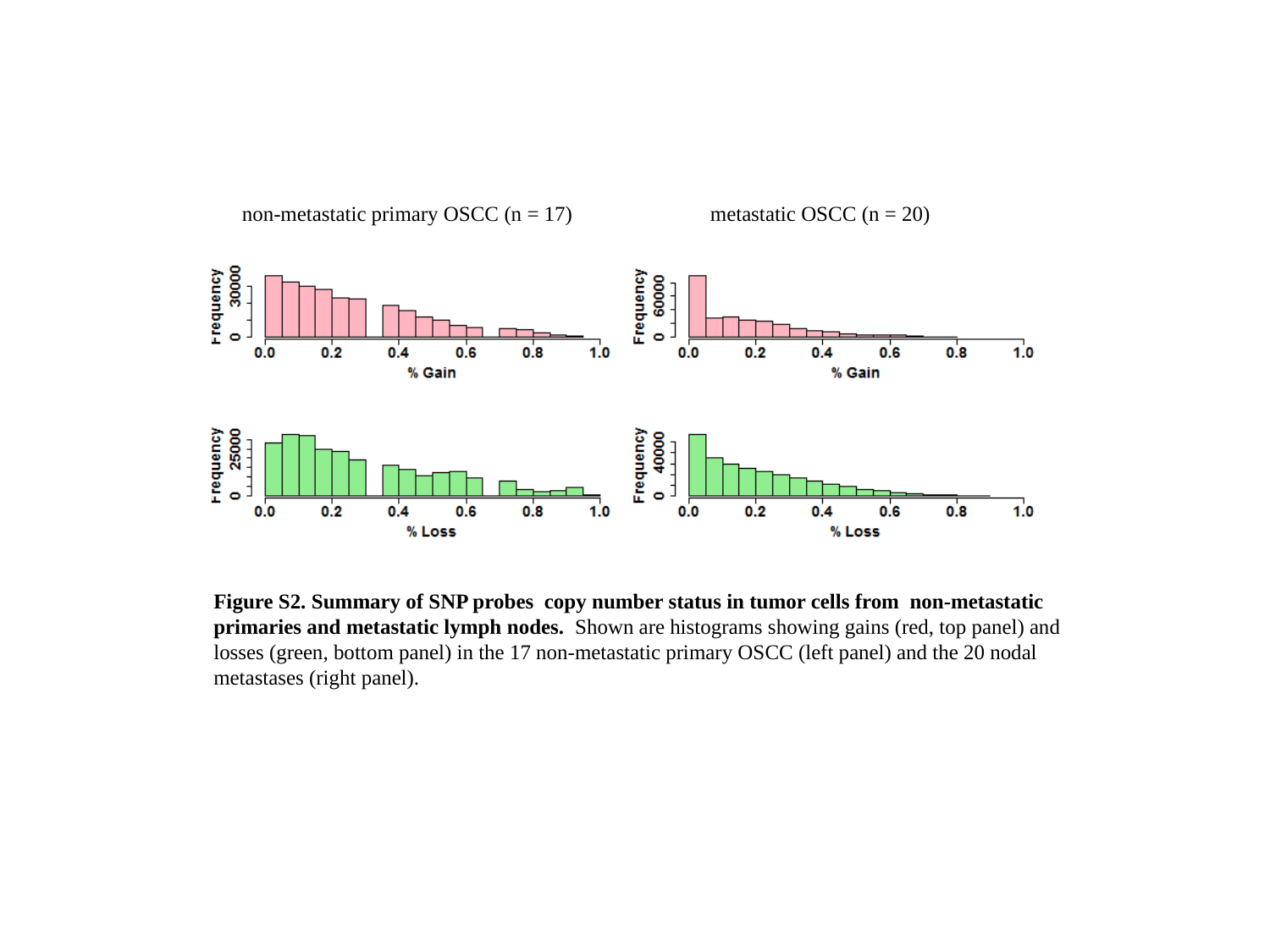

non-metastatic primary OSCC (n = 17)
metastatic OSCC (n = 20)
Figure S2. Summary of SNP probes copy number status in tumor cells from non-metastatic primaries and metastatic lymph nodes. Shown are histograms showing gains (red, top panel) and losses (green, bottom panel) in the 17 non-metastatic primary OSCC (left panel) and the 20 nodal metastases (right panel).
