# Supplementary material for: Integrative Genomics in Combination with RNA Interference Identifies Prognostic and Functionally Relevant Gene Targets for Oral Squamous Cell Carcinoma
Source: PLoS Genet. 2013 Jan 17;9(1):e1003169. doi: 10.1371/journal.pgen.1003169 (PMC3547824; doi:10.1371/journal.pgen.1003169)
Supplement: Figure S3 — Association between DNA copy number-associated differentially expressed genes and survival in the validation dataset excluding HPV+ oropharyngeal carcinomas. A) Hierarchical clustering of the 133-patient dataset excluding the HPV+ oropharyngeal carcinomas using the 95 CNA-associated differentially-expressed gene signature. Expression variances of the genes were summarized by principal components analysis. A hierarchical clustering analysis was performed using the first three principal component (PC) scores; B) Overall survival of the two patient clusters stratified by the 95-CNA-associated differentially expressed signature; C) Cumulative incidence of OSCC-specific death for the two patient clusters. (PPTX) [file pgen.1003169.s003.pptx]

## Slide 1
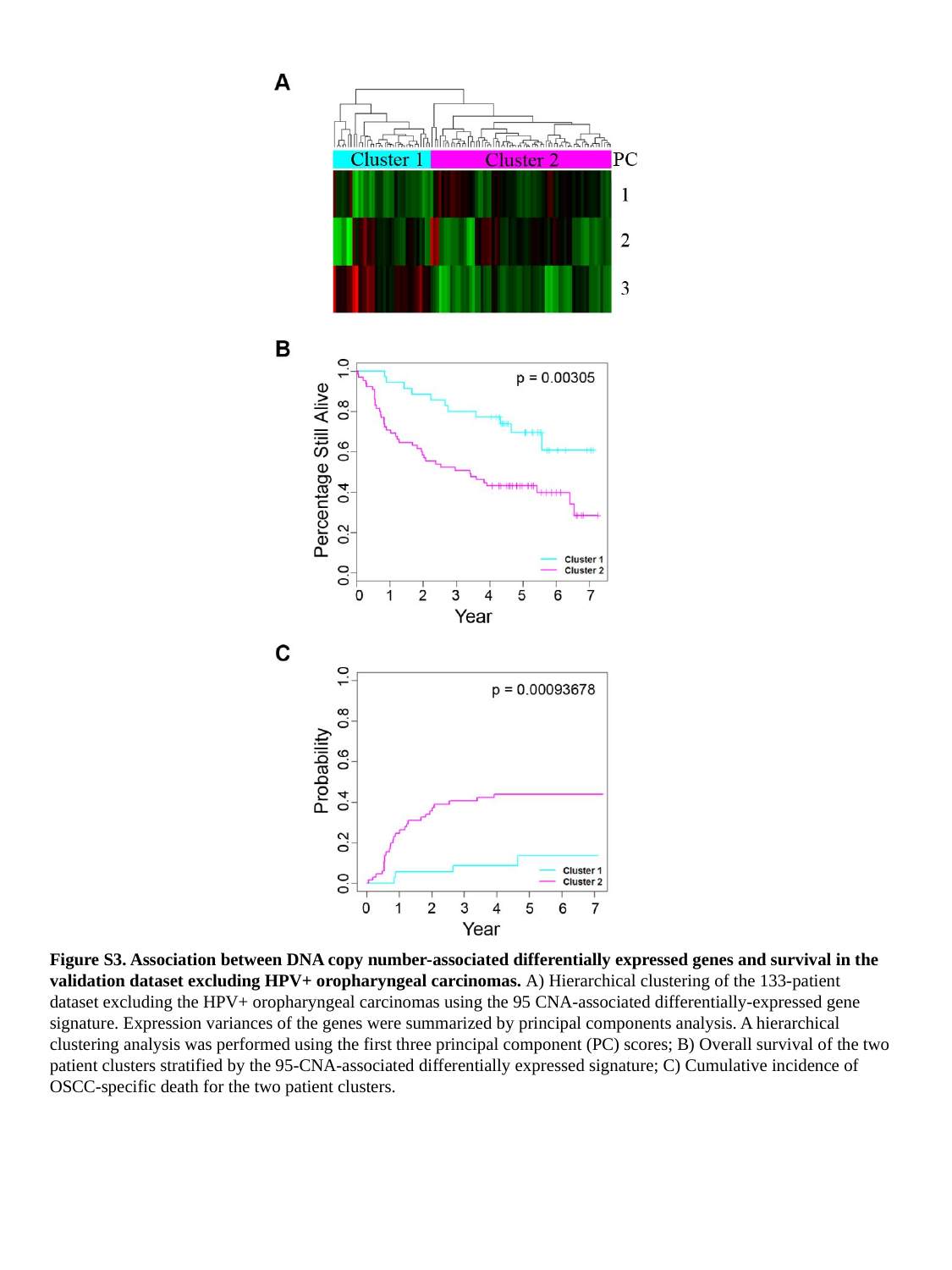

Figure S3. Association between DNA copy number-associated differentially expressed genes and survival in the validation dataset excluding HPV+ oropharyngeal carcinomas. A) Hierarchical clustering of the 133-patient dataset excluding the HPV+ oropharyngeal carcinomas using the 95 CNA-associated differentially-expressed gene signature. Expression variances of the genes were summarized by principal components analysis. A hierarchical clustering analysis was performed using the first three principal component (PC) scores; B) Overall survival of the two patient clusters stratified by the 95-CNA-associated differentially expressed signature; C) Cumulative incidence of OSCC-specific death for the two patient clusters.
