# Supplementary material for: Integrative Genomics in Combination with RNA Interference Identifies Prognostic and Functionally Relevant Gene Targets for Oral Squamous Cell Carcinoma
Source: PLoS Genet. 2013 Jan 17;9(1):e1003169. doi: 10.1371/journal.pgen.1003169 (PMC3547824; doi:10.1371/journal.pgen.1003169)
Supplement: Figure S4 — Characterization of cell migration capability of OSCC cell lines by wound-healing assay. Cells were plated into 6-well plates at a density of 1×10^6^/well. A sterile 200 µl pipette tip was used to scratch the cells to form a wound. The cells were then washed twice with 1×PBS and maintained in medium with 0.1% FBS. Pictures were taken at the 0, 8 and 20 hrs. Given the fast rate of wound closure in the JHU-019-SCC line, pictures were taken at 0, 3, 6 and 12 hrs. (A) Representative images show cell migration (wound-healing) at 0, 8, and 20 hr. time points for UM-SCC14A and 14C lines. (B) Cell migration rate was measured using ImageJ software (Wayne Rasband, NIH, Bethesda, MD). Results are present as the average percentage of wound-healing rate from 3 wounds. Error bars represent the standard deviation. (PPTX) [file pgen.1003169.s004.pptx]

## Slide 1
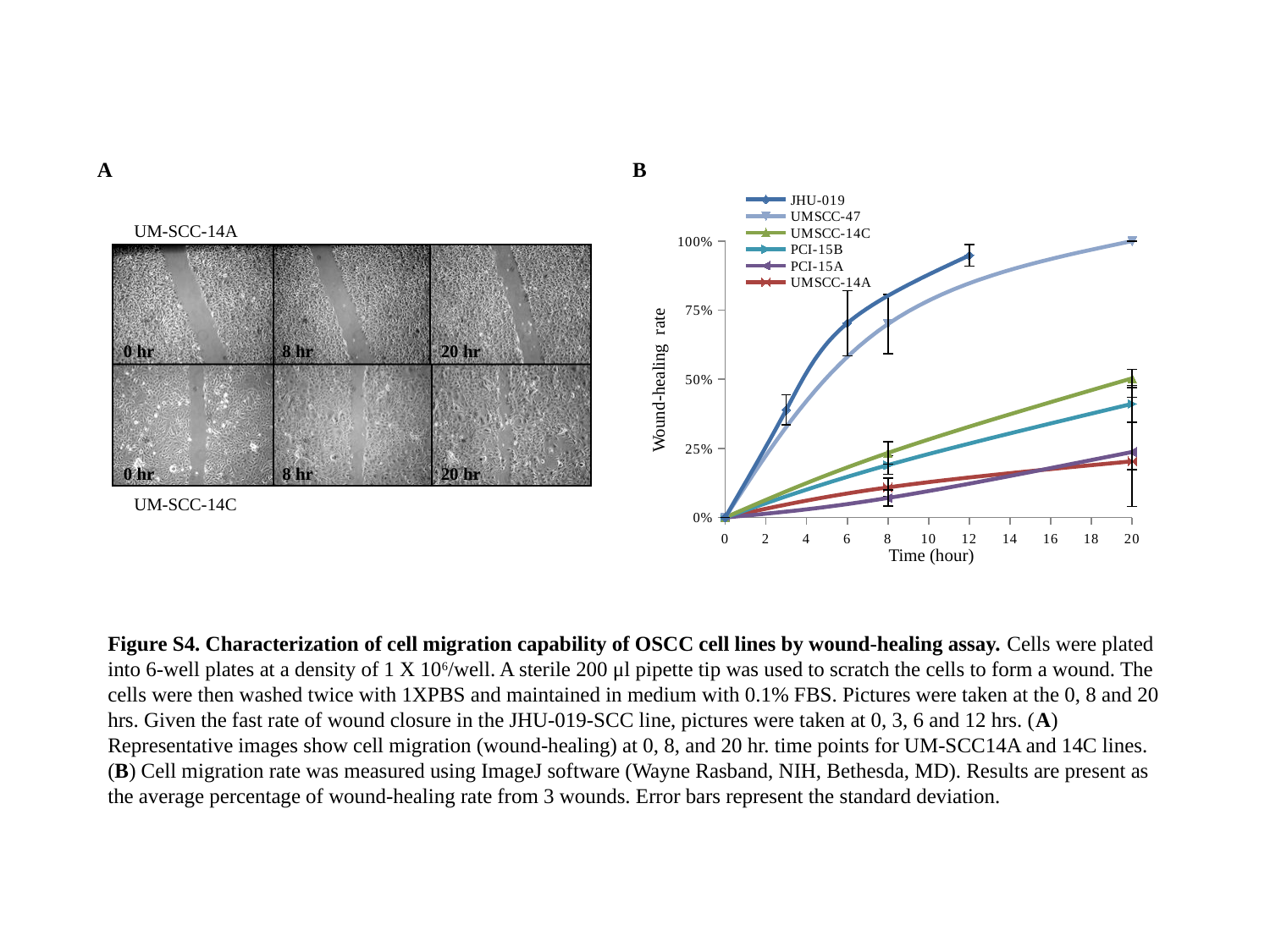

A
B
### Chart
| Category | JHU-019 | UMSCC-47 | UMSCC-14C | PCI-15B | PCI-15A | UMSCC-14A |
|---|---|---|---|---|---|---|UM-SCC-14A
Wound-healing rate
0 hr
8 hr
20 hr
0 hr
8 hr
20 hr
UM-SCC-14C
Time (hour)
Figure S4. Characterization of cell migration capability of OSCC cell lines by wound-healing assay. Cells were plated into 6-well plates at a density of 1 X 106/well. A sterile 200 μl pipette tip was used to scratch the cells to form a wound. The cells were then washed twice with 1XPBS and maintained in medium with 0.1% FBS. Pictures were taken at the 0, 8 and 20 hrs. Given the fast rate of wound closure in the JHU-019-SCC line, pictures were taken at 0, 3, 6 and 12 hrs. (A) Representative images show cell migration (wound-healing) at 0, 8, and 20 hr. time points for UM-SCC14A and 14C lines. (B) Cell migration rate was measured using ImageJ software (Wayne Rasband, NIH, Bethesda, MD). Results are present as the average percentage of wound-healing rate from 3 wounds. Error bars represent the standard deviation.
