# Supplementary material for: Integrative Genomics in Combination with RNA Interference Identifies Prognostic and Functionally Relevant Gene Targets for Oral Squamous Cell Carcinoma
Source: PLoS Genet. 2013 Jan 17;9(1):e1003169. doi: 10.1371/journal.pgen.1003169 (PMC3547824; doi:10.1371/journal.pgen.1003169)
Supplement: Figure S5 — Anchorage dependent and independent growth rates of OSCC cell lines. Growth rates with and without anchorage dependence were determined using standard 96-well tissue culture plates (A) and the plates coated with Poly-HEMA (B). The growth rates were measured with CellTiter-Glo Luminescent Cell Viability Assay kit (Promega, Madison, WI). The growth rates are presented as the average percentage of growth relative to day 0 from 3 repeated wells and error bars represent the standard deviation. (PPTX) [file pgen.1003169.s005.pptx]

## Slide 1
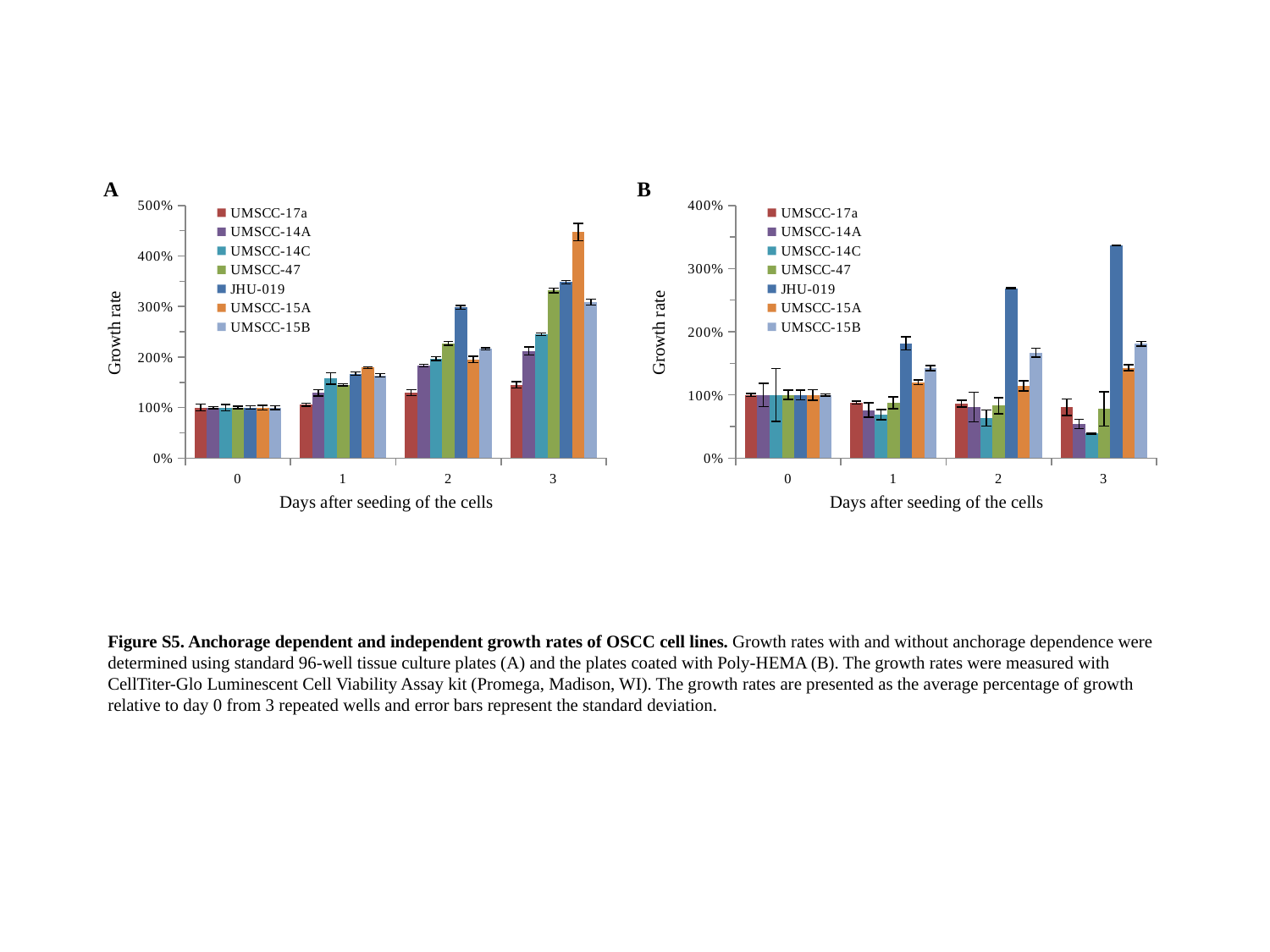

A
B
### Chart
| Category | UMSCC-17a | UMSCC-14A | UMSCC-14C | UMSCC-47 | JHU-019 | UMSCC-15A | UMSCC-15B |
|---|---|---|---|---|---|---|---|
| | 1.0 | 1.0 | 1.0 | 1.0 | 1.0 | 1.0 | 1.0 |
| | 1.0553959165243376 | 1.2939731158728207 | 1.5775994441197019 | 1.446982876761732 | 1.670307931169101 | 1.7903050695352114 | 1.636910068468173 |
| | 1.2945262116480099 | 1.830718627761622 | 1.9704790029657941 | 2.2688672985098313 | 2.980748846610737 | 1.9545418182671206 | 2.1632907920145463 |
| | 1.4467924791508162 | 2.1214115689022757 | 2.450465917462427 | 3.3197285093983058 | 3.4815317224643834 | 4.470566404758024 | 3.084247321165914 |
### Chart
| Category | UMSCC-17a | UMSCC-14A | UMSCC-14C | UMSCC-47 | JHU-019 | UMSCC-15A | UMSCC-15B |
|---|---|---|---|---|---|---|---|
| | 1.0 | 1.0 | 1.0 | 1.0 | 1.0 | 1.0 | 1.0 |
| | 0.878555632578651 | 0.7603922392628042 | 0.6890083595730107 | 0.8725880307091818 | 1.81428670618769 | 1.2020393939564065 | 1.4276415636870232 |
| | 0.8610865591821796 | 0.8101940858481269 | 0.6339995342668248 | 0.8305964142246388 | 2.689071224034401 | 1.1444241888099602 | 1.667905963234609 |
| | 0.8057899921499213 | 0.5404346453295459 | 0.3910682164106822 | 0.7800156178412956 | 3.3653936858940594 | 1.4306202635299925 | 1.8110864337114716 |Growth rate
Growth rate
Days after seeding of the cells
Days after seeding of the cells
Figure S5. Anchorage dependent and independent growth rates of OSCC cell lines. Growth rates with and without anchorage dependence were determined using standard 96-well tissue culture plates (A) and the plates coated with Poly-HEMA (B). The growth rates were measured with CellTiter-Glo Luminescent Cell Viability Assay kit (Promega, Madison, WI). The growth rates are presented as the average percentage of growth relative to day 0 from 3 repeated wells and error bars represent the standard deviation.
