# Supplementary material for: Integrative Genomics in Combination with RNA Interference Identifies Prognostic and Functionally Relevant Gene Targets for Oral Squamous Cell Carcinoma
Source: PLoS Genet. 2013 Jan 17;9(1):e1003169. doi: 10.1371/journal.pgen.1003169 (PMC3547824; doi:10.1371/journal.pgen.1003169)
Supplement: Figure S6 — Boxplots showing growth suppression effects of siRNA knockdown experiments targeting the 26 vs. the 708 gene set. Growth suppression was defined as ≥30% loss in cell viability in at least one cell line and labeled as “Minimum Survival Rate” in the y-axis. The boxplots show the median and the first and third quartile range (IQR) of the Minimun Survival Rate. The whiskers show the minimum and maximum values that are within 1.5× the IQR, and the dots beyond the whisker represent the outliers defined as values beyond Q3+1.5XIQR or Q1-1.5XIQR. (PPTX) [file pgen.1003169.s006.pptx]

## Slide 1
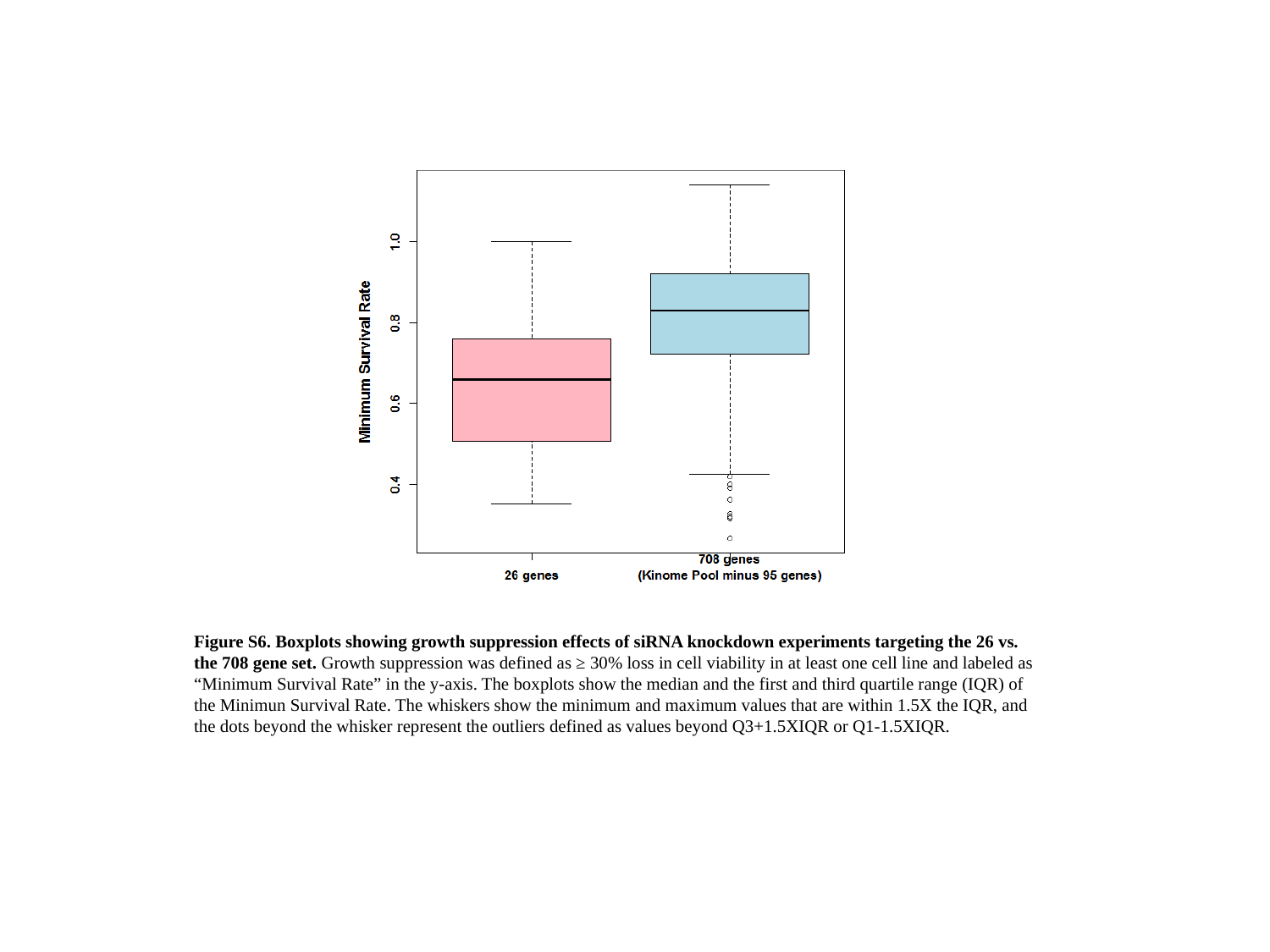

Figure S6. Boxplots showing growth suppression effects of siRNA knockdown experiments targeting the 26 vs. the 708 gene set. Growth suppression was defined as ≥ 30% loss in cell viability in at least one cell line and labeled as “Minimum Survival Rate” in the y-axis. The boxplots show the median and the first and third quartile range (IQR) of the Minimun Survival Rate. The whiskers show the minimum and maximum values that are within 1.5X the IQR, and the dots beyond the whisker represent the outliers defined as values beyond Q3+1.5XIQR or Q1-1.5XIQR.
