# Supplementary material for: Integrative Genomics in Combination with RNA Interference Identifies Prognostic and Functionally Relevant Gene Targets for Oral Squamous Cell Carcinoma
Source: PLoS Genet. 2013 Jan 17;9(1):e1003169. doi: 10.1371/journal.pgen.1003169 (PMC3547824; doi:10.1371/journal.pgen.1003169)
Supplement: Figure S7 — G3BP1 knockdown and Ras activity. The protein levels of G3BP1, phosphorylated and total Ras, phosphorylated and total Erk1, 2 in PCI-15B are shown at 48 hr and 72 hr after G3BP1 KD. Beta-actin is used as a loading control. (PPTX) [file pgen.1003169.s007.pptx]

## Slide 1
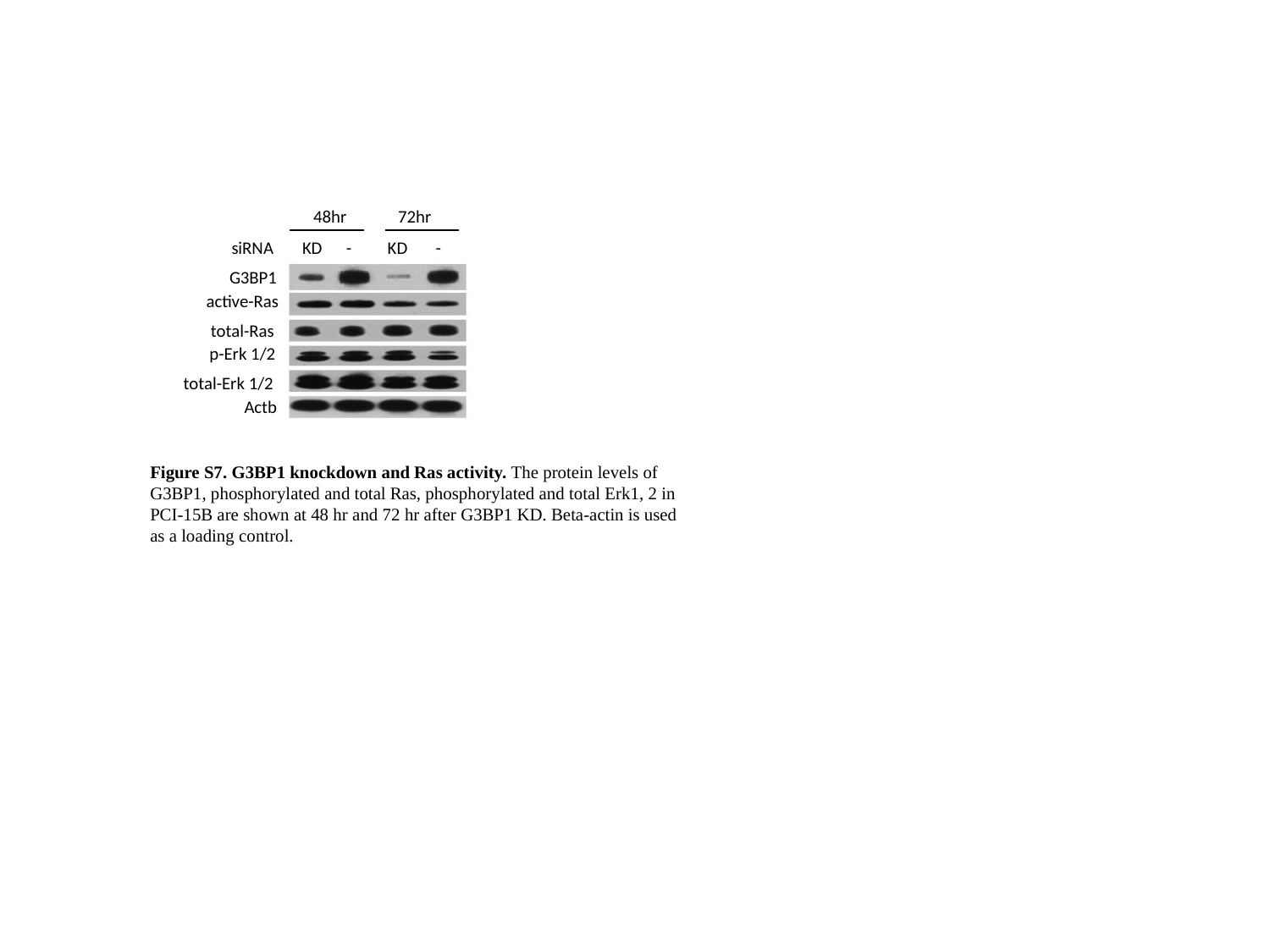

48hr
72hr
siRNA
KD - KD -
G3BP1
active-Ras
total-Ras
p-Erk 1/2
 total-Erk 1/2
 Actb
Figure S7. G3BP1 knockdown and Ras activity. The protein levels of G3BP1, phosphorylated and total Ras, phosphorylated and total Erk1, 2 in PCI-15B are shown at 48 hr and 72 hr after G3BP1 KD. Beta-actin is used as a loading control.
