# Supplementary material for: Integrative Genomics in Combination with RNA Interference Identifies Prognostic and Functionally Relevant Gene Targets for Oral Squamous Cell Carcinoma
Source: PLoS Genet. 2013 Jan 17;9(1):e1003169. doi: 10.1371/journal.pgen.1003169 (PMC3547824; doi:10.1371/journal.pgen.1003169)
Supplement: Table S2 — Ingenuity Pathway Analysis top functional categories of the CNA-associated differentially expressed genes. (PPTX) [file pgen.1003169.s009.pptx]

## Slide 1
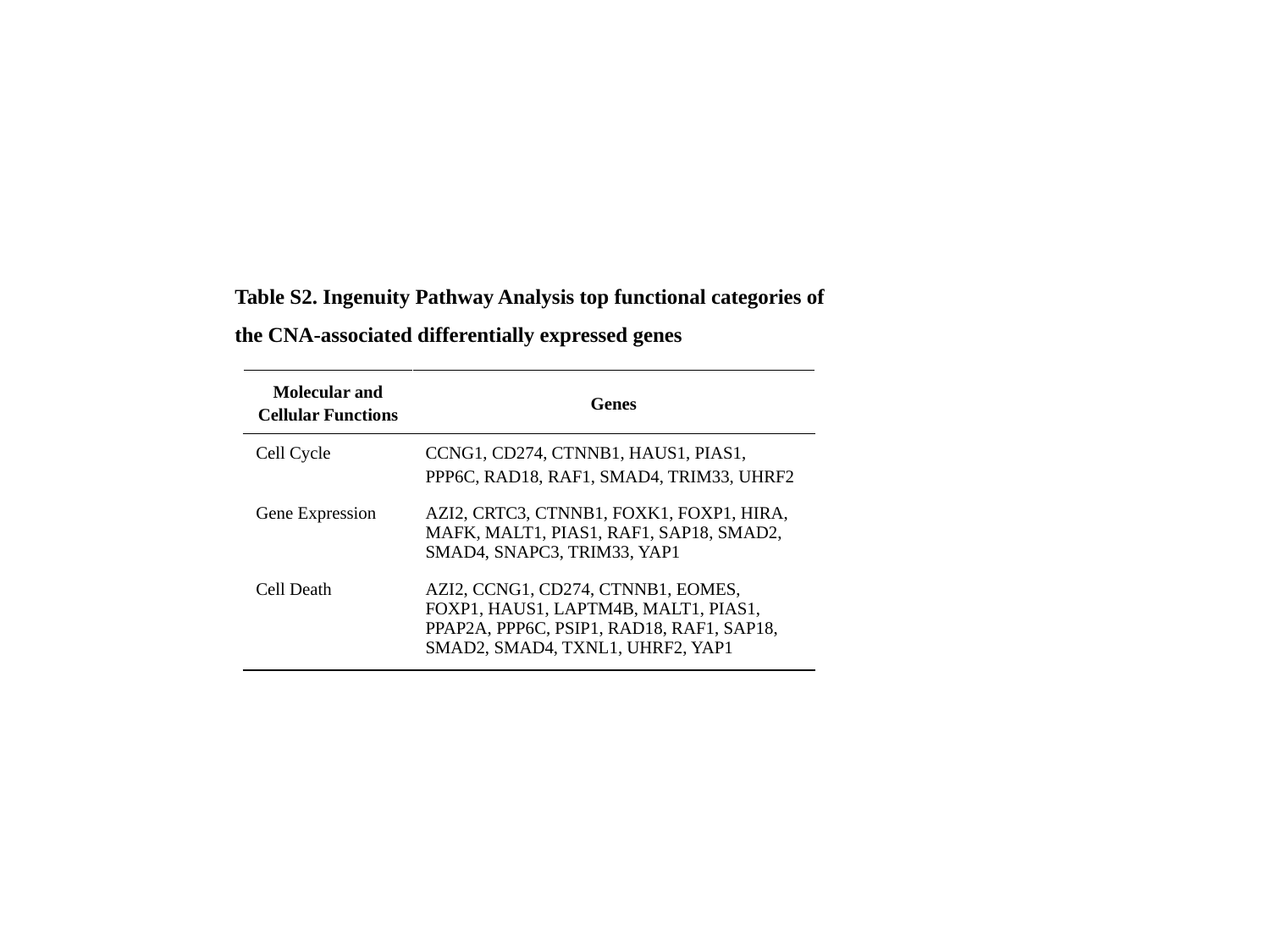

Table S2. Ingenuity Pathway Analysis top functional categories of the CNA-associated differentially expressed genes
| Molecular and Cellular Functions | Genes |
| --- | --- |
| Cell Cycle | CCNG1, CD274, CTNNB1, HAUS1, PIAS1, PPP6C, RAD18, RAF1, SMAD4, TRIM33, UHRF2 |
| Gene Expression | AZI2, CRTC3, CTNNB1, FOXK1, FOXP1, HIRA, MAFK, MALT1, PIAS1, RAF1, SAP18, SMAD2, SMAD4, SNAPC3, TRIM33, YAP1 |
| Cell Death | AZI2, CCNG1, CD274, CTNNB1, EOMES, FOXP1, HAUS1, LAPTM4B, MALT1, PIAS1, PPAP2A, PPP6C, PSIP1, RAD18, RAF1, SAP18, SMAD2, SMAD4, TXNL1, UHRF2, YAP1 |
