# Supplementary material for: Integrative Genomics in Combination with RNA Interference Identifies Prognostic and Functionally Relevant Gene Targets for Oral Squamous Cell Carcinoma
Source: PLoS Genet. 2013 Jan 17;9(1):e1003169. doi: 10.1371/journal.pgen.1003169 (PMC3547824; doi:10.1371/journal.pgen.1003169)
Supplement: Table S4 — Characteristics of the OSCC cell lines used for siRNA functional screens. (PPTX) [file pgen.1003169.s011.pptx]

## Slide 1
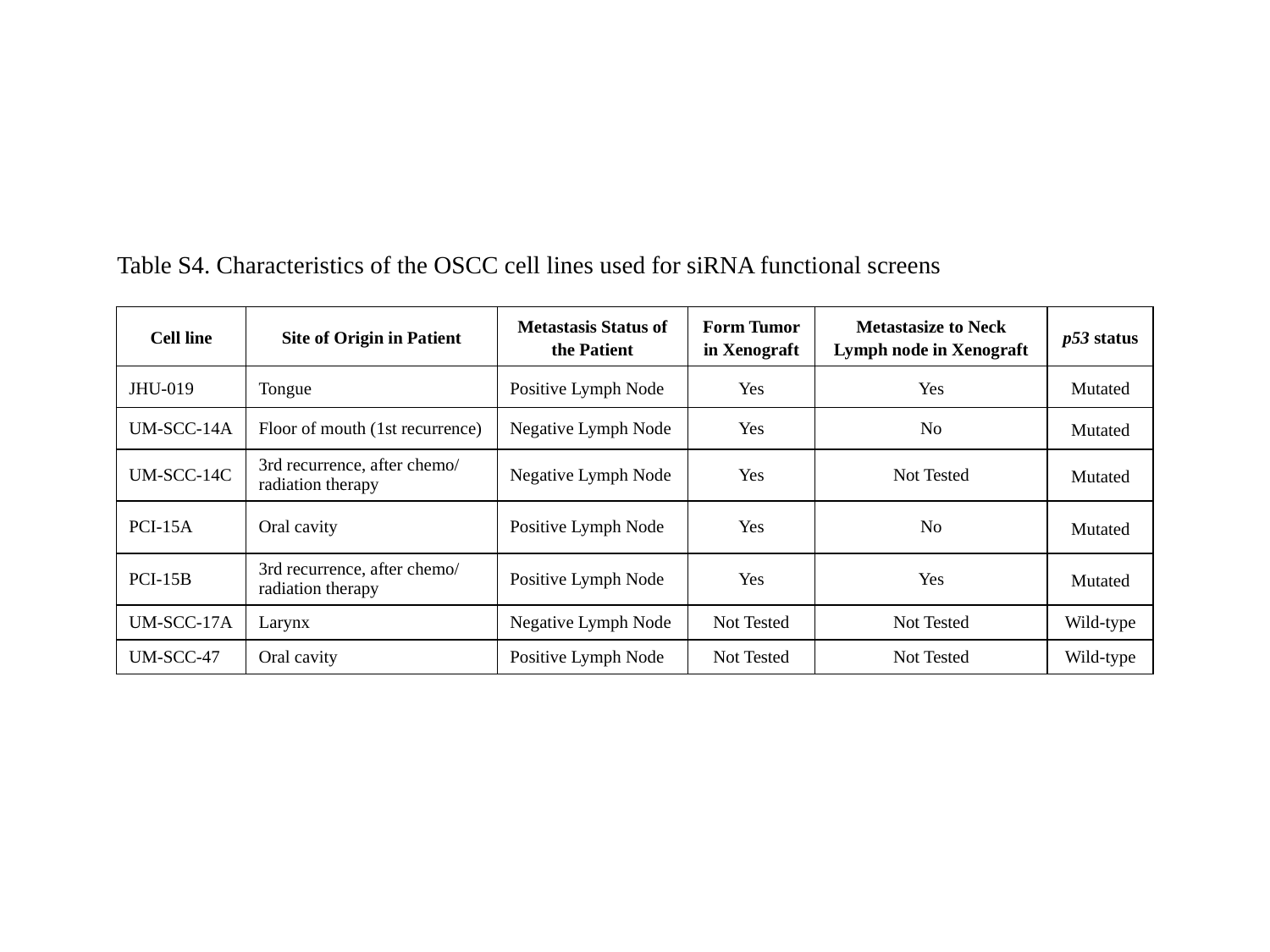

Table S4. Characteristics of the OSCC cell lines used for siRNA functional screens
| Cell line | Site of Origin in Patient | Metastasis Status of the Patient | Form Tumor in Xenograft | Metastasize to Neck Lymph node in Xenograft | p53 status |
| --- | --- | --- | --- | --- | --- |
| JHU-019 | Tongue | Positive Lymph Node | Yes | Yes | Mutated |
| UM-SCC-14A | Floor of mouth (1st recurrence) | Negative Lymph Node | Yes | No | Mutated |
| UM-SCC-14C | 3rd recurrence, after chemo/ radiation therapy | Negative Lymph Node | Yes | Not Tested | Mutated |
| PCI-15A | Oral cavity | Positive Lymph Node | Yes | No | Mutated |
| PCI-15B | 3rd recurrence, after chemo/ radiation therapy | Positive Lymph Node | Yes | Yes | Mutated |
| UM-SCC-17A | Larynx | Negative Lymph Node | Not Tested | Not Tested | Wild-type |
| UM-SCC-47 | Oral cavity | Positive Lymph Node | Not Tested | Not Tested | Wild-type |
